# Supplementary material for: Detection of haplotype-dependent allele-specific DNA methylation in WGBS data
Source: Nat Commun. 2020 Oct 16;11:5238. doi: 10.1038/s41467-020-19077-1 (PMC7567826; doi:10.1038/s41467-020-19077-1)
Supplement: Supplementary file 1 — Supplementary Information [file 41467_2020_19077_MOESM1_ESM.pdf]

# Supplementary Information

## Detection of haplotype-dependent allele-specific DNA methylation in WGBS data

Abante et al., 2020

# Supplementary Methods

## 1 Existing methods for ASM analysis

**Nonparametric independent method.** The methylation state within an allele that contains $N$ CpG sites $n = 1, 2, \ldots, N$ can be modeled by using the $N \times 1$ stochastic methylation state vector $\mathbf{X} = [X_1, X_2, \ldots, X_N]^T$, where $X_n = 0$ if the $n$-th CpG site is unmethylated and $X_n = 1$ if it is methylated. An early model for ASM analysis[1,2] assumes that CpG methylation occurs in a statistically independent manner. This implies that the probability distribution of methylation (PDM) is given by

$$p(\mathbf{x}) = \Pr[\mathbf{X} = \mathbf{x}] = \prod_{n=1}^{N} q_n(x_n), \quad \text{for every } \mathbf{x} \in \{0,1\}^N, \tag{1}$$

with

$$q_n(x_n) = \Pr[X_n = x_n] = \pi_n^{x_n}(1 - \pi_n)^{1-x_n}, \tag{2}$$

where $\pi_n = \Pr[X_n = 1]$ is the probability that the $n$-th CpG site is methylated. Note that we use here capital letters to denote random variables/vectors and small letters to denote their observed values.

Evaluation of the methylation probability $p(\mathbf{x})$ requires knowledge of the probabilities $\pi_n$, for $n = 1, 2, \ldots, N$. These probabilities can be estimated from available WGBS data using a maximum-likelihood approach that solves the following optimization problem:

$$\{\widehat{\pi}_1, \widehat{\pi}_2, \ldots, \widehat{\pi}_N\} = \arg \max_{\{\pi_1, \pi_2, \ldots, \pi_N\}} \ln p(\mathbf{x}_1, \mathbf{x}_2, \ldots, \mathbf{x}_M), \tag{3}$$

where $p(\mathbf{x}_1, \mathbf{x}_2, \ldots, \mathbf{x}_M)$ is the probability of observing $M$ independent (and possibly partial) reads $\{\mathbf{x}_1, \mathbf{x}_2, \ldots, \mathbf{x}_M\}$ of the allele's full methylation state. From Supplementary Equations (1) and (2), note that

$$\begin{aligned} p(\mathbf{x}_1, \mathbf{x}_2, \ldots, \mathbf{x}_M) &= \Pr[\mathbf{X} = \mathbf{x}_1, \mathbf{X} = \mathbf{x}_2, \ldots, \mathbf{X} = \mathbf{x}_M] \\ &= \prod_{m=1}^{M} \Pr[\mathbf{X} = \mathbf{x}_m] \\ &= \prod_{m=1}^{M} \prod_{n=1}^{N} \pi_n^{x_{m,n}} (1 - \pi_n)^{x_{m,n}} \end{aligned}$$

$$= \prod_{n=1}^{N} \pi_n^{\sum_{m=1}^{M} x_{m,n}} (1 - \pi_n)^{\sum_{m=1}^{M} x_{m,n}}$$

$$= \prod_{n=1}^{N} \pi_n^{M_n} (1 - \pi_n)^{U_n}, \tag{4}$$

where $M_n$ and $U_n$ are the numbers of reads in which the $n$-th CpG site is methylated and unmethylated, respectively. Supplementary Equations (3) and (4) lead to an empirical estimate $\widehat{\pi}_n$ of the probability $\pi_n$, given by

$$\widehat{\pi}_n = \frac{M_n}{M_n + U_n}, \quad \text{for } n = 1, 2, \ldots, N, \tag{5}$$

which in turn provides the following estimates $\widehat{q}_n(x_n)$ and $\widehat{p}(\mathbf{x})$ of the methylation probabilities $q_n(x_n)$ and $p(\mathbf{x})$:

$$\widehat{q}_n(x_n) = \left(\frac{M_n}{M_n + U_n}\right)^{x_n} \left(\frac{U_n}{M_n + U_n}\right)^{1-x_n}, \quad \text{for every } x_n, \tag{6}$$

and

$$\widehat{p}(\mathbf{x}) = \prod_{n=1}^{N} \left(\frac{M_n}{M_n + U_n}\right)^{x_n} \left(\frac{U_n}{M_n + U_n}\right)^{1-x_n}, \quad \text{for every } \mathbf{x}. \tag{7}$$

Due to the independence assumption, we refer to the ASM analysis method that uses the previous approach as the nonparametric independent (NPI) method. Note, however, that the independence assumption underlying this method seems unrealistic, given the known processivity of the DNMT enzymes[3–5] and the fact that analysis of WGBS data reveal extensive cooperativity in methylation[6]. Moreover, reliable estimation of the methylation probabilities $\pi_n$ that is consistent under experimental replication requires that the number of available observations at each CpG site is much larger than the number of methylation states (i.e., $M_n + U_n \gg 2$, for every $n$), which may not always be achievable when using WGBS data.

**Nonparametric dependent method.** In a recent paper[7], an approach was proposed by Onuchic et al. for modeling the methylation state within a homologous allele that does not impose the assumption of statistical independence. Given $M$ independent reads $\{\mathbf{x}_1, \mathbf{x}_2, \ldots, \mathbf{x}_M\}$ of the full methylation state within the allele, this approach calculates the maximum likelihood estimate $\widehat{p}(\mathbf{x})$ of the methylation probability $p(\mathbf{x})$ by solving the following constrained optimization

problem

$$
\begin{aligned}
\{\widehat{p}(\mathbf{x})\} &= \arg\max_{p} \ln p(\mathbf{x}_1, \mathbf{x}_2, \ldots, \mathbf{x}_M) \\
&= \arg\max_{p} \ln \prod_{\mathbf{x}} [p(\mathbf{x})]^{M(\mathbf{x})} \\
&= \arg\max_{p} \sum_{\mathbf{x}} M(\mathbf{x}) \ln p(\mathbf{x}), \qquad (8)
\end{aligned}
$$

subject to

$$\sum_{\mathbf{x}} p(\mathbf{x}) = 1, \qquad (9)$$

where $M(\mathbf{x})$ is the number of reads in which the methylation state within the allele is $\mathbf{x}$. This leads to an empirical estimate $\widehat{p}(\mathbf{x})$ of the probability $p(\mathbf{x})$, given by

$$\widehat{p}(\mathbf{x}) = \begin{cases} M(\mathbf{x}) / \sum_{\mathbf{x}'} M(\mathbf{x}'), & \text{if } \mathbf{x} \in \{\mathbf{x}_1, \mathbf{x}_2, \ldots, \mathbf{x}_M\} \\ 0, & \text{otherwise.} \end{cases} \qquad (10)$$

Since the previous approach does not assume statistical independence between the methylation states at individual CpG sites, we refer to the ASM analysis method that uses this approach as the nonparametric dependent (NPD) method. Note, however, that performing ASM analysis using the NPD method requires reads that correspond to fully observed methylation states. Moreover, to produce reliable estimates of the methylation probabilities, this approach requires that the number of such reads is much larger than the number of methylation states (i.e., $M \gg 2^N$), which is not possible using current WGBS technologies when considering alleles containing many CpG sites. For this reason, the analysis by Onuchic et al. is limited to genomic regions that contain $4$ CpG sites, which are referred to as epialleles.

## 2 A Potential energy function for ASM analysis

By invoking the well-known maximum-entropy principle[8], we can show that the probability distribution of the methylation state $\mathbf{X}$ that is consistent with methylation means and pair-wise correlations at each CpG site of a genomic region that contains $N$ CpG sites $1, 2, \ldots, N$, is given by

$$p(\mathbf{x}) = \frac{1}{Z} \exp\{-U(\mathbf{x})\}, \text{ for every } \mathbf{x} \in \{0,1\}^N, \qquad (11)$$

where

$$U(\mathbf{x}) = -\sum_{n=1}^{N} a_n(2x_n - 1) - \sum_{n=1}^{N-1} b_n(2x_n - 1)(2x_{n+1} - 1) \qquad (12)$$

is the potential energy function, $a_n$ and $b_n$ are two parameters associated with the $n$-th CpG site, and

$$Z = \sum_{\mathbf{x}} \exp\{-U(\mathbf{x})\} \tag{13}$$

is a normalizing constant, known as the partition function.

In a previous work by Jenkinson et al.[6,9], the goal was to perform DNA methylation analysis along the entire genome using the potential energy function given by Supplementary Equation (12). However, reliable estimation of its $2N$ parameters $a$ and $b$ from available WGBS data is not feasible since, for large $N$, such estimation requires the availability of a large amount of data, which is not possible with current WGBS technology. This problem was previously addressed by Jenkinson e al. by partitioning the genome into non-overlapping estimation regions of 3-kb each and by setting, within the $k$-th region,

$$a_n = c_{k,1} + c_{k,2}\rho_n \quad \text{and} \quad b_n = c_{k,3}/d_n, \tag{14}$$

where $c_{k,1}$, $c_{k,2}$, and $c_{k,3}$ are three parameters characteristic to the $k$-th estimation region, $\rho_n$ is the CpG density at site $n$, and $d_n$ is the distance between CpG sites $n$ and $n+1$. This approach reduced the problem of parameter estimation to estimating only the three parameters $c_{k,1}$, $c_{k,2}$, and $c_{k,3}$ within the $k$-th estimation region, as opposed to estimating $2N$ parameters.

It turns out that, even when employing the Supplementary Equations (14), performing methylation analysis using Supplementary Equations (11)-(13) is highly problematic, since computing key statistical quantities within a genomic region of interest requires evaluation of partition functions and marginalizing probabilities based on computationally intensive formulas, as well as extensive Monte Carlo estimation, which take an unrealistic amount of time even on a high performance computer cluster. There are two underlying reasons for this problem: ($i$) the combinatorial complexity of the methylation state-space, which grows geometrically with $N$ (the state-space contains $2^N$ methylation patterns for a genomic region with $N$ CpG sites), and ($ii$) the dependence of the $a$ and $b$ parameters of the potential energy function on the CpG sites. These issues were previously addressed by Jenkinson et al.[6,9] by partitioning the genome into non-overlapping genomic units, composed of 150 bp each, and by performing methylation analysis at the resolution of one genomic unit. This led to characterizing DNA methylation using the probability distribution of the methylation level within a genomic unit (i.e., the average of all methylation states at individual CpG sites within the genomic unit), which resulted in a computationally manageable approach to genome-wide methylation analysis.

In contrast to the above, the goal in this paper is to perform hap-ASM analysis by identifying significant differences between the distributions of methylation patterns, and associated statistics that correspond to the homologous alleles of a given haplotype. This requires computation of the probabilities of individual methylation patterns (e.g., 1111100000 and 0000011111 within an allele containing $10$ CpG sites), as compared to the previous method by Jenkinson et al.[6,9], which instead uses the probabilities of the methylation level within small genomic units. This distinction does not seem to be an issue when dealing with small genomic units containing a few CpG sites (e.g., within a genomic unit of 150 bp containing $4$ CpG sites the difference between methylation patterns 1100 and 0011 may not be of significant importance since they both have the same methylation level of 0.5 and the CpG sites are located in close proximity to each other). However, it is quite important when dealing with large haplotypes (e.g., within a homologous allele containing $10$ CpG sites, the difference between patterns 1111100000 and 0000011111, which have the same methylation level of 0.5, may be significant, considering the fact that some CpG sites may not be located close to each other). For this reason, the previous approach to methylation analysis by Jenkinson et al. is not appropriate for hap-ASM analysis.

Due to limitations in read-based phasing, which is often constrained by the length of WGS reads and the low coverage of WGBS, current sequencing technologies only allow analysis of relatively small haplotypes (for example, the size of more than $99\%$ of the haplotypes analyzed in the real data was no more than 1-kb). Given the small amount of available WGBS data, it is reasonable to assume that we can accurately observe only the average of the methylation means at individual CpG sites within small subregions of a given allele. Moreover, since pair-wise correlations are second-order moments, requiring more data for reliable estimation than the means, we can also assume that we can accurately observe only the average of these correlations at individual CpG sites within the entire allele. For these reasons, we subdivide each allele into a minimum number $K$ of equally sized non-overlapping subregions of size no more than $G$. Given the previous constraints, and by invoking the maximum-entropy principle, we can show that the potential energy function associated with the probability distribution of the methylation state $\mathbf{X}$ within a given allele that is consistent with the average of the methylation means within each allelic subregion and the average of the pair-wise correlations within the entire allele is given by

$$U(\mathbf{x}) = -\sum_{k=1}^{K} \alpha_k \sum_{n \in \mathcal{N}_k} (2x_n - 1) - \beta \sum_{n=1}^{N-1} (2x_n - 1)(2x_{n+1} - 1), \tag{15}$$

where $\mathcal{N}_k$ is the set of all CpG sites within the $k$-th allelic subregion, $\alpha_k$ is a parameter char-

acteristic to the $k$-th allelic subregion, and $\beta$ is a parameter characteristic to the entire allele. Parameter $\alpha_k$ influences the propensity of CpG sites in the $k$-th allelic subregion $\mathcal{R}_k$ to be methylated due to non-cooperative factors, while $\beta$ accounts for the fact that the methylation status of two contiguous CpG sites $n$ and $n+1$ within the allelic region $\mathcal{R}$ is most often highly correlated due to the known processivity of the DNMT enzymes[3–5]. Notably, the previous energy function given by Supplementary Equation (15), when used in conjunction with Supplementary Equations (11) and (13), does not imply equal mean methylation levels and pair-wise correlations at each CpG site of an allele, which can be shown to depend on the CpG site $n$.

The previous energy function leads to a coarse-grained version of the one in Supplementary Equation (12) which allows the CPEL method to perform hap-ASM analysis using a computationally feasible method that employs probability distributions of the methylation state, instead of the probability distributions of the methylation level utilized by Jenkinson et al.[6,9]. In subsequent sections, we show that computations in this case can be performed efficiently by multiplying $2 \times 2$ matrices evaluated by spectral decompositions, which require eigenvalues and eigenvectors that are calculated by analytical formulas, as well as by employing standard derivative approximations and a limited number of Monte Carlo estimations. Consequently, the CPEL method can perform hap-ASM analysis in the original space of individual methylation patterns, which allows this approach to identify types of significant allele-specific methylation imbalances inaccessible to current approaches.

The potential energy function associated with the CPEL method leads to a version of the well-known one-dimensional Ising model of statistical physics[10] characterized by a single interaction parameter $\beta$ and an external field parameter $\alpha$ that is not necessarily constant but depends on the specific subregion of a given allele. This simple choice enjoys several advantages: ($i$) its estimation from data involves computing the values of at most $K+1$ parameters, which can be done reliably using current WGBS technologies, ($ii$) when $\beta \neq 0$, it accounts for correlations in methylation at contiguous CpG sites, and ($iii$) it allows for the derivation of analytical formulas for necessary calculations, which can then be performed in a computationally efficient manner. Note that, when $\beta = 0$, the CPEL model is reduced to the NPI model. Moreover, as sequencing technology improves producing larger read sizes and coverage, the potential energy function given by Supplementary Equation (15) can be modified to better handle large haplotypes by allowing the interaction parameter $\beta$ to also depend on the specific subregion of a given allele.

Using the CPEL model requires that we determine a value for parameter $G$ (in bp). This value directly affects the granularity of modeling due to the dependence of the energy function

on the $K+1$ parameters $\alpha_1, \alpha_2, \ldots, \alpha_K$, and $\beta$, a number that increases with decreasing $G$. Since successful estimation of an increasing number of parameters requires more data in general, we expect the number of haplotypes analyzed by the CPEL method in a given sample to decrease with smaller values of $G$. This observation leads to the following scheme for determining an appropriate value for $G$ that strikes a balance between a finer model description of the methylation state and the number of haplotypes analyzed by the CPEL method using this value.

Procedure for determining the modeling granularity $G$

1. Compute the minimum integer $j_0$ such that $G = j_0 \times 100$ bp implies $K = 1$ for all alleles in a given sample, as well as the number $n_{j_0}$ of haplotypes analyzed by the CPEL method with the computed value of $G$ by counting all pairs of homologous alleles for which parameter estimation was successful in both alleles. Set $j = j_0 - 1$.
2. Set $G \leftarrow G - 100$ and determine the number $n_j$ of haplotypes analyzed by the CPEL method with the new value of $G$.
3. If $(n_{j_0} - n_j)/n_{j_0} \leq 0.05$, then set $j \leftarrow j - 1$ and go to Step 2.
4. If $(n_{j_0} - n_j)/n_{j_0} > 0.05$, then STOP and use the current value of $G$ when analyzing the sample.

This scheme determines the finest possible model description of the methylation state (i.e., the smallest possible value of $G$) that leads to no more than a $5\%$ loss in the number of haplotypes analyzed by the CPEL method in a given sample when comparing to the case of maximum model granularity. Notably, by applying this strategy on the real data, we consistently found that $G = 500$ bp in all samples.

## 3 Computing the partition function

Let us first consider the case when $K = 1$. In this case, the potential energy given by Supplementary Equation (15) becomes

$$U(\mathbf{x}) = -\alpha_1 \sum_{n=1}^{N} (2x_n - 1) - \beta \sum_{n=1}^{N-1} (2x_n - 1)(2x_{n+1} - 1), \tag{16}$$

where $N$ is the number of CpG sites in an allelic region $\mathcal{R}$. We now have that

$$
\begin{aligned}
Z &= \sum_{\mathbf{x}} \exp\{-U(\mathbf{x})\} \\
&= \sum_{\mathbf{x}} \exp\big\{\frac{\alpha_1}{2}(2x_1-1)\big\} \\
&\quad \times \left(\prod_{n=1}^{N-1} \exp\left\{\frac{\alpha_1}{2}(2x_n-1)+\beta(2x_n-1)(2x_{n+1}-1)+\frac{\alpha_1}{2}(2x_{n+1}-1)\right\}\right) \\
&\quad \times \exp\left\{\frac{\alpha_1}{2}(2x_N-1)\right\}. \qquad (17)
\end{aligned}
$$

If we set

$$
\mathbf{u}_1 = \begin{bmatrix} e^{-\alpha_1/2} \\ e^{\alpha_1/2} \end{bmatrix}, \qquad (18)
$$

and

$$
\mathbf{W}_1 = \begin{bmatrix} e^{\beta-\alpha_1} & e^{-\beta} \\ e^{-\beta} & e^{\beta+\alpha_1} \end{bmatrix}, \qquad (19)
$$

then Supplementary Equation (17) becomes

$$
Z = \mathbf{u}_1^T \mathbf{W}_1^{N-1} \mathbf{u}_1. \qquad (20)
$$

Now, let us consider the case when $K = 2$. In this case,

$$
U(\mathbf{x}) = -\alpha_1 \sum_{n=1}^{N_1}(2x_n-1) - \alpha_2 \sum_{n=N_1+1}^{N_1+N_2}(2x_n-1) - \beta \sum_{n=1}^{N_1+N_2-1}(2x_n-1)(2x_{n+1}-1), \qquad (21)
$$

where $N_1$ and $N_2$ respectively denote the numbers of CpG sites in the two subregions $\mathcal{R}_1$ and $\mathcal{R}_2$ of $\mathcal{R}$. We now have that

$$
\begin{aligned}
Z &= \sum_{\mathbf{x}} \exp\{-U(\mathbf{x})\} \\
&= \sum_{\mathbf{x}} \exp\big\{\frac{\alpha_1}{2}(2x_1-1)\big\} \\
&\quad \times \left(\prod_{n=1}^{N_1-1} \exp\left\{\frac{\alpha_1}{2}(2x_n-1)+\beta(2x_n-1)(2x_{n+1}-1)+\frac{\alpha_1}{2}(2x_{n+1}-1)\right\}\right) \\
&\quad \times \exp\left\{\frac{\alpha_1}{2}(2x_{N_1}-1)+\beta(2x_{N_1}-1)(2x_{N_1+1}-1)+\frac{\alpha_2}{2}(2x_{N_1+1}-1)\right\} \\
&\quad \times \left(\prod_{n=N_1+1}^{N_1+N_2-1} \exp\left\{\frac{\alpha_2}{2}(2x_n-1)+\beta(2x_n-1)(2x_{n+1}-1)+\frac{\alpha_2}{2}(2x_{n+1}-1)\right\}\right) \\
&\quad \times \exp\left\{\frac{\alpha_2}{2}(2x_{N_1+N_2}-1)\right\}. \qquad (22)
\end{aligned}
$$

If we set

$$\mathbf{u}_2 = \begin{bmatrix} e^{-\alpha_2/2} \\ e^{\alpha_2/2} \end{bmatrix}, \tag{23}$$

$$\mathbf{W}_2 = \begin{bmatrix} e^{\beta-\alpha_2} & e^{-\beta} \\ e^{-\beta} & e^{\beta+\alpha_2} \end{bmatrix}, \tag{24}$$

and

$$\mathbf{V}_2 = \begin{bmatrix} e^{\beta-(\alpha_1+\alpha_2)/2} & e^{-\beta-(\alpha_1-\alpha_2)/2} \\ e^{-\beta+(\alpha_1-\alpha_2)/2} & e^{\beta+(\alpha_1+\alpha_2)/2} \end{bmatrix}, \tag{25}$$

then Supplementary Equation (22) becomes

$$Z = \mathbf{u}_1^T \mathbf{W}_1^{N_1-1} \mathbf{V}_2 \mathbf{W}_2^{N_2-1} \mathbf{u}_2. \tag{26}$$

By following similar steps, we can show in general that

$$Z = \mathbf{u}_1^T \mathbf{W}_1^{N_1-1} \left( \prod_{k=2}^{K} \mathbf{V}_k \mathbf{W}_k^{N_k-1} \right) \mathbf{u}_K, \quad \text{for } K \geq 2, \tag{27}$$

where $N_k$ is the number of CpG sites within the $k$-th subregion $\mathcal{R}_k$ of $\mathcal{R}$,

$$\mathbf{u}_k = \begin{bmatrix} e^{-\alpha_k/2} \\ e^{\alpha_k/2} \end{bmatrix}, \tag{28}$$

$$\mathbf{W}_k = \begin{bmatrix} e^{\beta-\alpha_k} & e^{-\beta} \\ e^{-\beta} & e^{\beta+\alpha_k} \end{bmatrix}, \tag{29}$$

and

$$\mathbf{V}_k = \begin{bmatrix} e^{\beta-(\alpha_{k-1}+\alpha_k)/2} & e^{-\beta-(\alpha_{k-1}-\alpha_k)/2} \\ e^{-\beta+(\alpha_{k-1}-\alpha_k)/2} & e^{\beta+(\alpha_{k-1}+\alpha_k)/2} \end{bmatrix}. \tag{30}$$

The expression in Supplementary Equation (27) provides a formula for efficiently computing the partition function using $2 \times 2$ matrix multiplications. This formula however requires calculating powers $\mathbf{W}_k^{N_k-1}$ of matrix $\mathbf{W}_k$, which can be computationally inefficient for large values of $N_k$. If $\mathbf{W}_k = \mathbf{E}_k \mathbf{\Lambda}_k \mathbf{E}_k^T$ is the spectral decomposition of $\mathbf{W}_k$, where the columns of $\mathbf{E}_k$ are the (orthonormal) eigenvectors $\mathbf{e}_{k,1}$, $\mathbf{e}_{k,2}$ of $\mathbf{W}_k$, and $\mathbf{\Lambda}_k$ is a diagonal matrix with entries the corresponding eigenvalues $\lambda_{k,1}$, $\lambda_{k,2}$, then

$$\mathbf{W}_k^{N_k-1} = \mathbf{E}_k \mathbf{\Lambda}_k^{N_k-1} \mathbf{E}_k^T$$
$$= \lambda_{k,1}^{N_k-1} \mathbf{e}_{k,1} \mathbf{e}_{k,1}^T + \lambda_{k,2}^{N_k-1} \mathbf{e}_{k,2} \mathbf{e}_{k,2}^T, \quad (31)$$

which provides a formula for efficiently computing $\mathbf{W}_k^{N_k-1}$. It can be verified that

$$\lambda_{k,1} = e^{\beta} \cosh \alpha_k - e^{-\beta} \sqrt{1 + e^{4\beta} (\sinh \alpha_k)^2} \quad (32)$$

$$\lambda_{k,2} = e^{\beta} \cosh \alpha_k + e^{-\beta} \sqrt{1 + e^{4\beta} (\sinh \alpha_k)^2}, \quad (33)$$

with corresponding (orthonormal) eigenvectors given by

$$\mathbf{e}_{k,1} = \frac{1}{\sqrt{1 + \left[ e^{2\beta} \sinh \alpha_k + \sqrt{1 + e^{4\beta} (\sinh \alpha_k)^2} \right]^2}} \begin{bmatrix} e^{2\beta} \sinh \alpha_k + \sqrt{1 + e^{4\beta} (\sinh \alpha_k)^2} \\ -1 \end{bmatrix} \quad (34)$$

$$\mathbf{e}_{k,2} = \frac{1}{\sqrt{1 + \left[ e^{2\beta} \sinh \alpha_k - \sqrt{1 + e^{4\beta} (\sinh \alpha_k)^2} \right]^2}} \begin{bmatrix} e^{2\beta} \sinh \alpha_k - \sqrt{1 + e^{4\beta} (\sinh \alpha_k)^2} \\ -1 \end{bmatrix}. \quad (35)$$

## 4 Marginalizing methylation probabilities

In general, methylation reads are subject to missing information. Suppose that, in a given read $\mathbf{x}$ of the methylation state of an allele that is partitioned into $K$ subregions $\mathcal{R}_k$, $k = 1, 2, \ldots, K$, the methylation status of the last $m_{k_1}$ CpG sites within subregion $\mathcal{R}_{k_1}$ is not observed, and the same is true for the first $m_{k_1+1}$ CpG sites of the next subregion $\mathcal{R}_{k_1+1}$. Let $p_1$ and $q_1$ be the indices that determine the position of the first and the last unobserved CpG sites in the methylation vector $\mathbf{x}$, respectively, and let $\widetilde{\mathbf{x}}$ be the observed portion of $\mathbf{x}$. Then, the likelihood of observing $\widetilde{\mathbf{x}}$ is obtained by marginalizing the probability $\Pr[\mathbf{X} = \mathbf{x}]$ of the methylation state $\mathbf{X}$ over the unobserved CpG sites, leading to

$$\Pr[\widetilde{\mathbf{X}} = \widetilde{\mathbf{x}}] = \frac{1}{Z} \sum_{\{x_{p_1}, \ldots, x_{q_1}\}} \exp \left\{ \sum_{k=1}^{K} \alpha_k \sum_{n \in \mathcal{N}_k} (2x_n - 1) + \beta \sum_{n=1}^{N-1} (2x_n - 1)(2x_{n+1} - 1) \right\}$$
$$= \frac{e^{-U_0(\widetilde{\mathbf{x}})}}{Z} \sum_{\{x_{p_1}, \ldots, x_{q_1}\}} \exp \left\{ \alpha_{k_1} \sum_{n=p_1}^{p_1+m_{k_1}-1} (2x_n - 1) + \alpha_{k_1+1} \sum_{n=p_1+m_{k_1}}^{q_1} (2x_n - 1) \right.$$
$$\left. + \beta \sum_{n=p_1-1}^{q_1} (2x_n - 1)(2x_{n+1} - 1) \right\}, \quad (36)$$

with

$$U_0(\widetilde{\mathbf{x}}) = -\sum_{k=1}^{K} \alpha_k \sum_{n \in \mathcal{N}'_k} (2x_n - 1) - \beta \sum_{n \in \mathcal{N}''} (2x_n - 1)(2x_{n+1} - 1), \tag{37}$$

where $\mathcal{N}'_k$ is the set of CpG sites within subregion $\mathcal{R}_k$ of the allele other than $\{p_1, p_1+1, \ldots, q_1\}$ and $\mathcal{N}''$ is the set of CpG sites within the entire allele other than $\{p_1 - 1, p_1, \ldots, q_1\}$.

Note now that

$$\sum_{\{x_{p_1},\ldots,x_{q_1}\}} \exp\left\{ \alpha_{k_1} \sum_{n=p_1}^{p_1+m_{k_1}-1} (2x_n - 1) + \alpha_{k_1+1} \sum_{n=p_1+m_{k_1}}^{q_1} (2x_n - 1) + \beta \sum_{n=p_1-1}^{q_1} (2x_n - 1)(2x_{n+1} - 1) \right\}$$
$$= \sum_{\{x_{p_1},\ldots,x_{q_1}\}} \exp\left\{ \left[\frac{\alpha_{k_1}}{2} + \beta(2x_{p_1-1} - 1)\right](2x_{p_1} - 1) \right\}$$
$$\times \prod_{n=p_1}^{p_1+m_{k_1}-2} \left[ \exp\left\{ \frac{\alpha_{k_1}}{2}(2x_n - 1) + \beta(2x_n - 1)(2x_{n+1} - 1) + \frac{\alpha_{k_1}}{2}(2x_{n+1} - 1) \right\} \right]$$
$$\times \exp\left\{ \frac{\alpha_{k_1}}{2}(2x_{p_1+m_{k_1}-1} - 1) + \beta x_{p_1+m_{k_1}-1} x_{p_1+m_{k_1}} + \frac{\alpha_{k_1+1}}{2}(2x_{p_1+m_{k_1}} - 1) \right\}$$
$$\times \prod_{n=p_1+m_{k_1}}^{q_1-1} \left[ \exp\left\{ \frac{\alpha_{k_1+1}}{2}(2x_n - 1) + \beta(2x_n - 1)(2x_{n+1} - 1) + \frac{\alpha_{k_1+1}}{2}(2x_{n+1} - 1) \right\} \right]$$
$$\times \exp\left\{ \left[\frac{\alpha_{k_1+1}}{2} + \beta(2x_{q_1+1} - 1)\right](2x_{q_1} - 1) \right\}. \tag{38}$$

If we set

$$\mathbf{u}_k(x) = \begin{bmatrix} e^{-\alpha_k/2 - \beta_k(2x-1)} \\ e^{\alpha_k/2 + \beta_k(2x-1)} \end{bmatrix}, \tag{39}$$

then

$$\sum_{\{x_{p_1},\ldots,x_{q_1}\}} \exp\left\{ \alpha_{k_1} \sum_{n=p_1}^{p_1+m_{k_1}-1} (2x_n - 1) + \alpha_{k_1+1} \sum_{n=p_1+m_{k_1}}^{q_1} (2x_n - 1) + \beta \sum_{n=p_1-1}^{q_1} (2x_n - 1)(2x_{n+1} - 1) \right\}$$
$$= [\mathbf{u}_{k_1}(x_{p_1-1})]^T \mathbf{W}_{k_1}^{m_{k_1}-1} \mathbf{V}_{k_1+1} \mathbf{W}_{k_1+1}^{m_{k_1+1}-1} \mathbf{u}_{k_1+1}(x_{q_1+1}), \tag{40}$$

where the matrices $\mathbf{W}_k$ and $\mathbf{V}_k$ are given by Supplementary Equations (29) and (30), respectively. As a result, we obtain

$$\Pr[\widetilde{\mathbf{X}} = \widetilde{\mathbf{x}}] = \frac{e^{-U_0(\widetilde{\mathbf{x}})}}{Z} [\mathbf{u}_{k_1}(x_{p_1-1})]^T \mathbf{W}_{k_1}^{m_{k_1}-1} \mathbf{V}_{k_1+1} \mathbf{W}_{k_1+1}^{m_{k_1+1}-1} \mathbf{u}_{k_1+1}(x_{q_1+1}), \tag{41}$$

where $U_0(\widetilde{\mathbf{x}})$ is given by Supplementary Equation (37). Note that the potential energy $U_0(\widetilde{\mathbf{x}})$ in this expression is defined over the observed portion of $\mathbf{x}$ and is similar to the one given by Supplementary Equation (15). Moreover, the vector $\mathbf{u}_{k_1}(x_{p_1-1})$ takes into account the portion of the potential energy at the boundary between the observed and unobserved parts of $\mathcal{R}_{k_1}$, whereas the vector $\mathbf{u}_{k_1+1}(x_{q_1+1})$ takes into account the portion of the potential energy at the boundary between the unobserved and observed parts of $\mathcal{R}_{k_1+1}$. Finally, the matrix product term $\mathbf{W}_{k_1}^{m_{k_1}-1}\mathbf{V}_{k_1+1}\mathbf{W}_{k_1+1}^{m_{k_1+1}-1}$ accounts for the marginalization of the methylation states over the unobserved CpG sites $p_1, p_1+1, \ldots, q_1$. Note also that powers of the matrices $\mathbf{W}_{k_1}$ and $\mathbf{W}_{k_1+1}$ can be efficiently computed using the spectral decomposition approach employed for computing the partition function.

More generally, if the methylation status of the last $m_{k_1}$ CpG sites within subregion $\mathcal{R}_{k_1}$ and the first $m_{k_1+1}$ CpG sites of subregion $\mathcal{R}_{k_1+1}$ is not observed, and the same is true for the last $m_{k_2}$ CpG sites within another subregion $\mathcal{R}_{k_2}$ and the first $m_{k_2+1}$ CpG sites of subregion $\mathcal{R}_{k_2+1}$, where $k_2 \geq k_1 + 2$, then we can show that

$$\begin{aligned}\Pr[\widetilde{\mathbf{X}} = \widetilde{\mathbf{x}}] \;=\; & \frac{e^{-U_0(\widetilde{\mathbf{x}})}}{Z}[\mathbf{u}_{k_1}(x_{p_1-1})]^T \mathbf{W}_{k_1}^{m_{k_1}-1}\mathbf{V}_{k_1+1}\mathbf{W}_{k_1+1}^{m_{k_1+1}-1}\mathbf{u}_{k_1+1}(x_{q_1+1}) \\ & \times [\mathbf{u}_{k_2}(x_{p_2-1})]^T \mathbf{W}_{k_2}^{m_{k_2}-1}\mathbf{V}_{k_2+1}\mathbf{W}_{k_2+1}^{m_{k_2+1}-1}\mathbf{u}_{k_2+1}(x_{q_2+1}), \qquad (42)\end{aligned}$$

with $U_0(\widetilde{\mathbf{x}})$ given again by Supplementary Equation (37), where $p_1$ and $q_1$ are the indices that determine the position of the first and the last unobserved CpG sites within the region $\mathcal{R}_{k_1} \cup \mathcal{R}_{k_1+1}$, $p_2$ and $q_2$ are the indices that determine the position of the first and the last unobserved CpG sites within the region $\mathcal{R}_{k_2} \cup \mathcal{R}_{k_2+1}$, $\mathcal{N}'_k$ contains all CpG sites in subregion $\mathcal{R}_k$ of the allele other than $\{p_1, p_1+1, \ldots, q_1, p_2, p_2+1, \ldots, q_2\}$, and $\mathcal{N}''$ contains all CpG sites in the entire allele other than $\{p_1-1, p_1, \ldots, q_1, p_2-1, p_2, \ldots, q_2\}$. Note that the potential energy $U_0(\widetilde{\mathbf{x}})$ in this expression is again defined over the observed portion of $\mathbf{x}$ and is similar to the one given by Supplementary Equation (15). As before, the vector $\mathbf{u}_{k_1}(x_{p_1-1})$ takes into account the portion of the potential energy at the boundary between the observed and unobserved parts of $\mathcal{R}_{k_1}$, whereas the vector $\mathbf{u}_{k_1+1}(x_{q_1+1})$ takes into account the portion of the potential energy at the boundary between the unobserved and observed parts of $\mathcal{R}_{k_1+1}$. Moreover, the vector $\mathbf{u}_{k_2}(x_{p_2-1})$ takes into account the portion of the potential energy at the boundary between the observed and unobserved parts of $\mathcal{R}_{k_2}$, whereas the vector $\mathbf{u}_{k_2+1}(x_{q_2+1})$ takes into account the portion of the potential energy at the boundary between the unobserved and observed parts of $\mathcal{R}_{k_2+1}$. Finally, the term $\mathbf{W}_{k_1}^{m_{k_1}-1}\mathbf{V}_{k_1+1}\mathbf{W}_{k_1+1}^{m_{k_1+1}-1}$ accounts for the marginalization of the methylation states over the unobserved CpG sites $p_1, p_1+1, \ldots, q_1$, whereas the term $\mathbf{W}_{k_2}^{m_{k_2}-1}\mathbf{V}_{k_2+1}\mathbf{W}_{k_2+1}^{m_{k_2+1}-1}$

accounts for the marginalization of the methylation states over the unobserved CpG sites $p_2$, $p_2 + 1, \ldots, q_2$.

By following the previous steps and rules, we can also derive formulas for computing the probability $\Pr[\widetilde{\mathbf{X}} = \widetilde{\mathbf{x}}]$ of a methylation read for the general case in which a given read $\mathbf{x}$ of the methylation state of a given allele is subject to multiple stretches of unobserved CpG sites.

## 5 Performing maximum-likelihood estimation

The Supplementary Equation (15) defines a potential energy landscape $U(\mathbf{x})$, $\mathbf{x} \in \{0,1\}^N$, which is fully specified by the $K+1$ parameters $\alpha_1, \alpha_2, \ldots, \alpha_K$, and $\beta$. Since for subregions that do not contain any CpG sites the $\alpha$ parameters can be set equal to zero, it is expected that the number of parameters associated with most alleles to be less than $K+1$ and, therefore, the potential energy function involves a small number of parameters (at most $K+1$) that must be estimated from available WGBS data.

Given $M$ independent and fully observed reads $\mathbf{x}_1, \mathbf{x}_2, \ldots, \mathbf{x}_M$ of the methylation state in a given allele, obtained by properly mapping WGBS data to each identified allele (see Methods in the main paper), the CPEL method estimates the parameters $\boldsymbol{\theta} = [\alpha_1, \alpha_2, \ldots, \alpha_K, \beta]^T$ of the potential energy landscape by solving the following maximum-likelihood optimization problem

$$\widehat{\boldsymbol{\theta}} = \arg\max_{\boldsymbol{\theta}} \sum_{m=1}^{M} \ln p_{\boldsymbol{\theta}}(\mathbf{x}_m). \tag{43}$$

However, due to the relatively low coverage of WGBS data and other technical difficulties associated with this technology, any method for hap-ASM analysis must be designed to handle partial observations of the methylation state (i.e., observations in which the methylation state is not observed at all CpG sites). If the $m$-th read provides a partial observation $\widetilde{\mathbf{x}}_m$ of the methylation state, then the probability $p_{\boldsymbol{\theta}}(\mathbf{x}_m)$ in Supplementary Equation (43) is replaced with a probability computed from the former by marginalization (i.e., by summing over the methylation states of all unobserved CpG sites). Note, however, that the log-likelihood function will not be in general concave in this case, and maximum-likelihood estimation must be done by a non-convex global optimization algorithm.

To determine an appropriate algorithm for this task, we compared ten global optimization methods and found three of them to outperform the rest. We evaluated the performance of each algorithm by generating data from a CPEL model with known parameter values in the five cases depicted in Supplementary Table 8, by computing the time it took for convergence, by evaluating the error in parameter estimation, and by repeating this process 100 times with different

initializations. Particle Swarm[11], QuadDIRECT (https://github.com/timholy/QuadDIRECT.jl), a method inspired by DIRECT[12] and Multilevel Coordinate Search[13], as well as Simulated Annealing[14], were found to be the most accurate and fastest algorithms and selected these methods for a more refined comparison. Among the algorithms we discarded after the first comparison were Simultaneous Perturbation Stochastic Approximation[15], exponential Natural Evolution Strategies[16], and Direct Search[17].

During the second comparison, we tested Particle Swarm, QuadDIRECT with four different values for the maximum number of objective function evaluations, and Simulated Annealing with four different values for the temperature reduction factor $r$ (new temperature $= r \times$ old temperature). According to representative results depicted in Supplementary Figure 10, Particle Swarm (PS) and Simulated Annealing with $r = 10^{-4}$ (SA3) produced the best estimation performance in terms of the median and interquartile range (IQR) of the resulting errors, quantified by the Euclidean distance between the estimated parameter values and their true values. However, PS took substantially more time to converge, whereas all four versions of Simulated Annealing converged must faster than PS. Finally, some versions of QuadDIRECT converged faster than Simulated Annealing, but produced larger estimation errors. In light of these results, we chose SA3 for global optimization, which we implemented using the `Optim.jl` package[18] of `Julia`[19]. These observations, together with our parameter estimation results (see Supplementary Figure 2), which show that the use of SA3 recovers the true parameter values with increasing accuracy as more data become available, demonstrates the stability of SA3 for parameter estimation.

## 6 Homozygous and heterozygous haplotypes

Haplotypes are mostly homozygous, which means that there is a perfect match between the CpG sites in the two homologous alleles. However, a SNP may remove a CpG site from one of the two alleles or introduce a new CpG site. In this case, the haplotype is heterozygous, meaning that the CpG sites in its two homologous alleles will not match at certain genomic locations. Notably, about $20\%$ of the haplotypes in the data considered in this paper are heterozygous. Since homologous alleles in heterozygous haplotypes contain mismatched CpG sites, we cannot model their methylation using state vectors of the same dimensionality and, therefore, we cannot directly compare them in terms of their methylation. To address this issue, we describe methylation within an allele of a heterozygous haplotype using the vector $\widetilde{\mathbf{X}}$ of the methylation states at all CpG sites that are common to its homologous pair. We then characterize methylation by means of the probabilities $\Pr[\widetilde{\mathbf{X}} = \widetilde{\mathbf{x}}]$, which we compute by marginalizing the correspond-

ing estimated probabilities $\Pr[\mathbf{X} = \mathbf{x}]$ over the methylation states at all non-matching CpG sites using the method described in Supplementary Section 4.

## 7 Computing the mean methylation level

**Homozygous haplotypes.** Consider a homozygous allelic region $\mathcal{R}$ with $N$ CpG sites $n = 1, 2, \ldots, N$. From Eq. (7) in the main paper, note that

$$\begin{aligned} \mu(\mathbf{X}) &= \mathrm{E}\left[\frac{1}{N}\sum_{n=1}^{N} X_n\right] \\ &= \mathrm{E}\left[\frac{1}{2} + \frac{1}{2N}\sum_{n=1}^{N}(2X_n - 1)\right] \\ &= \frac{1}{2} + \frac{1}{2N}\mathrm{E}\left[\sum_{n=1}^{N}(2X_n - 1)\right] \\ &= \frac{1}{2} + \frac{1}{2N}\mathrm{E}\left[\sum_{k=1}^{K}\sum_{n\in\mathcal{N}_k}(2X_n - 1)\right] \\ &= \frac{1}{2} + \frac{1}{2N}\sum_{k=1}^{K}\mathrm{E}\left[\sum_{n\in\mathcal{N}_k}(2X_n - 1)\right], \end{aligned} \tag{44}$$

where $\mathcal{N}_k$ is the set of CpG sites within the subregion $\mathcal{R}_k$ of $\mathcal{R}$. From a known result shown by Bickel and Doksum[20] (Corollary 1.6.1), and since the CPEL model given by Eqs. (1)-(3) in the main paper is a canonical exponential family, we can show that

$$\mathrm{E}\left[\sum_{n\in\mathcal{N}_k}(2X_n - 1)\right] = \frac{\partial \ln Z}{\partial \alpha_k}, \quad \text{for } k = 1, 2, \ldots, K, \tag{45}$$

in which case

$$\mu(\mathbf{X}) = \frac{1}{2}\left(1 + \frac{1}{N}\sum_{k=1}^{K}\frac{\partial \ln Z}{\partial \alpha_k}\right). \tag{46}$$

This formula provides a fast method for evaluating the MML $\mu(\mathbf{X})$, which can be done by computing the partition function using the method discussed in Supplementary Section 3, as well as the derivatives of the logarithm of the partition function with respect to parameters $\alpha$ using a standard derivative approximation technique.

**Heterozygous haplotypes.** For a heterozygous allele with $M$ CpG sites $m = 1, 2, \ldots, M$ common to its homologous pair, we define the MML by

$$\mu(\widetilde{\mathbf{X}}) = \mathrm{E}\left[\frac{1}{M}\sum_{m=1}^{M}\widetilde{X}_m\right], \tag{47}$$

where $\widetilde{\mathbf{X}}$ is the vector of the methylation states at all common CpG sites. Since we compute the probabilities of $\widetilde{\mathbf{X}}$ from the estimated probabilities $\Pr[\mathbf{X} = \mathbf{x}]$ of the entire methylation state $\mathbf{X}$ within the heterozygous allele using marginalization (see Supplementary Section 4), this distribution will not in general be in a canonical exponential form and, therefore, the previous derivative-based method cannot be used to evaluate $\mu(\widetilde{\mathbf{X}})$. However,

$$\begin{aligned}\mu(\widetilde{\mathbf{X}}) &= \mathrm{E}\left[\frac{1}{M}\sum_{m=1}^{M}\widetilde{X}_m\right] = \frac{1}{M}\sum_{m=1}^{M}\mathrm{E}[\widetilde{X}_m] = \frac{1}{M}\sum_{m=1}^{M}\sum_{x=0,1} x\Pr[\widetilde{X}_m = x]\\ &= \frac{1}{M}\sum_{m=1}^{M}\Pr[\widetilde{X}_m = 1].\end{aligned} \tag{48}$$

This implies that we can evaluate the MML $\mu(\widetilde{\mathbf{X}})$ using Supplementary Equation (48), provided that we know the probabilities $\Pr[\widetilde{X}_m = 1]$, $m = 1, 2, \ldots, M$. We can directly compute these probabilities from the probabilities $\Pr[\mathbf{X} = \mathbf{x}]$ using marginalization (see Supplementary Section 4).

## 8 Computing the normalized methylation entropy

**Homozygous haplotypes** From Eqs. (1), (2), and (8) in the main paper and for a homozygous allelic region $\mathcal{R}$ with $N$ CpG sites $n = 1, 2, \ldots, N$, we have

$$\begin{aligned}h(\mathbf{X}) &= -\frac{1}{N}\sum_{\mathbf{x}} p(\mathbf{x})\log_2 p(\mathbf{x})\\ &= -\frac{1}{N\ln 2}\sum_{\mathbf{x}} p(\mathbf{x})\ln p(\mathbf{x})\\ &= -\frac{1}{N\ln 2}\left\{-\ln Z + \mathrm{E}\Big[\sum_{k=1}^{K}\alpha_k\sum_{n\in\mathcal{N}_k}(2X_n - 1) + \beta\sum_{n=1}^{N-1}(2X_n-1)(2X_{n+1}-1)\Big]\right\}\\ &= \frac{1}{N\ln 2}\left\{\ln Z - \sum_{k=1}^{K}\alpha_k\mathrm{E}\Big[\sum_{n\in\mathcal{N}_k}(2X_n - 1)\Big] - \beta\mathrm{E}\Big[\sum_{n=1}^{N-1}(2X_n-1)(2X_{n+1}-1)\Big]\right\}.\end{aligned} \tag{49}$$

Since the CPEL model given by Eqs. (1)-(3) in the main paper is a canonical exponential family, we can show, in addition to Supplementary Equation (45), that

$$\mathrm{E}\left[\sum_{n=1}^{N}(2X_n-1)(2X_{n+1}-1)\right] = \frac{\partial \ln Z}{\partial \beta}, \tag{50}$$

which, together with Supplementary Equation (49), leads to

$$h(\mathbf{X}) = \frac{1}{N\ln 2}\left\{\ln Z - \sum_{k=1}^{K}\alpha_k\frac{\partial \ln Z}{\partial \alpha_k} - \beta\frac{\partial \ln Z}{\partial \beta}\right\}. \tag{51}$$

Similarly to MML, this formula provides a fast method for evaluating the NME $h(\mathbf{X})$. This can be done by computing the partition function using the method discussed in Supplementary Section 3 and by evaluating the derivatives of the logarithm of the partition function with respect to the $\alpha$ and $\beta$ parameters using a standard derivative approximation technique.

**Heterozygous haplotypes.** For a heterozygous allele with $M$ CpG sites $m = 1, 2, \ldots, M$ common to its homologous pair, we define the NME by

$$h(\widetilde{\mathbf{X}}) = -\frac{1}{M}\sum_{\widetilde{\mathbf{x}}}\Pr[\widetilde{\mathbf{X}} = \widetilde{\mathbf{x}}]\log_2\Pr[\widetilde{\mathbf{X}} = \widetilde{\mathbf{x}}], \tag{52}$$

where $\widetilde{\mathbf{X}}$ is the vector of the methylation states at all common CpG sites. Similarly to the MML, we cannot use the previous derivative-based method to evaluate $h(\widetilde{\mathbf{X}})$. To illustrate how to compute $h(\widetilde{\mathbf{X}})$, consider an allelic region $\mathcal{R}$ that is partitioned into four subregions $\mathcal{R}_1$, $\mathcal{R}_2$, $\mathcal{R}_3$, $\mathcal{R}_4$, and assume that we are interested in computing the NME within three contiguous stretches of CpG sites spanning from CpG site 1 to CpG site $p-1$, from CpG site $q+1$ ($q \geq p+1$) to CpG site $p'-1$, and from to CpG site $q'+1$ ($q' \geq p'+1$) to CpG site $N$. Let us also assume that CpG sites $p, p+1, \ldots, q$ are contained within the first two subregions $\mathcal{R}_1$ and $\mathcal{R}_2$, with $\mathcal{R}_1$ containing $m_1$ of these sites and $\mathcal{R}_2$ containing $m_2$ sites, while CpG sites $p', p'+1, \ldots, q'$ are contained within subregions $\mathcal{R}_3$ and $\mathcal{R}_4$, with $\mathcal{R}_3$ containing $m_3$ sites and $\mathcal{R}_4$ containing $m_4$ sites. In this case, and by following similar arguments as the ones we used to show Supplementary Equations (37) and (42), we have that

$$\begin{aligned}\Pr[\widetilde{\mathbf{X}} = \widetilde{\mathbf{x}}] \;=\; & \frac{e^{-U_0(\widetilde{\mathbf{x}})}}{Z}[\mathbf{u}_1(x_{p-1})]^T\mathbf{W}_1^{m_1-1}\mathbf{V}_2\mathbf{W}_2^{m_2-1}\mathbf{u}_2(x_{q+1}) \\ & \times[\mathbf{u}_3(x_{p'-1})]^T\mathbf{W}_3^{m_3-1}\mathbf{V}_4\mathbf{W}_4^{m_4-1}\mathbf{u}_4(x_{q'+1}),\end{aligned} \tag{53}$$

with

$$U_0(\widetilde{\mathbf{x}}) = -\sum_{k=1}^{4}\alpha_k\sum_{n\in\mathcal{N}'_k}(2x_n-1) - \beta\sum_{n\in\mathcal{N}''}(2x_n-1)(2x_{n+1}-1), \tag{54}$$

where $\widetilde{\mathbf{X}}$ is the methylation state at CpG sites $1, 2, \ldots, p-1, q+1, \ldots, p'-1, q'+1, \ldots, N$, $\mathcal{N}'_k$ contains all CpG sites in subregion $\mathcal{R}_k$ of $\mathcal{R}$ other than $p, p+1, \ldots, q, p', p'+1, \ldots, q'$, and $\mathcal{N}''$ contains all CpG sites in $\mathcal{R}$ other than $p-1, p, \ldots, q, p'-1, p', \ldots, q'$. If we now set

$$g_1(X_{p-1}, X_{q+1}) = \ln \left( [\mathbf{u}_1(X_{p-1})]^T \mathbf{W}_1^{m_1-1} \mathbf{V}_2 \mathbf{W}_2^{m_2-1} \mathbf{u}_2(X_{q+1}) \right), \quad (55)$$

and

$$g_2(X_{p'-1}, X_{q'+1}) = \ln \left( [\mathbf{u}_3(X_{p'-1})]^T \mathbf{W}_3^{m_3-1} \mathbf{V}_4 \mathbf{W}_4^{m_4-1} \mathbf{u}_4(X_{q'+1}) \right), \quad (56)$$

then the NME of $\widetilde{\mathbf{X}}$ satisfies

$$\begin{aligned} h(\widetilde{\mathbf{X}}) &= -\frac{1}{L} \sum_{\widetilde{\mathbf{x}}} \Pr[\widetilde{\mathbf{X}} = \widetilde{\mathbf{x}}] \log_2 \Pr[\widetilde{\mathbf{X}} = \widetilde{\mathbf{x}}] \\ &= -\frac{1}{L \ln 2} \sum_{\widetilde{\mathbf{x}}} \Pr[\widetilde{\mathbf{X}} = \widetilde{\mathbf{x}}] \ln \Pr[\widetilde{\mathbf{X}} = \widetilde{\mathbf{x}}] \\ &= \frac{1}{L \ln 2} \Big( \ln Z + \mathrm{E}[U_0(\widetilde{\mathbf{X}})] - \mathrm{E}[g_1(X_{p-1}, X_{q+1})] - \mathrm{E}[g_2(X_{p'-1}, X_{q'+1})] \Big), \quad (57) \end{aligned}$$

with $L = N + p - q + p' - q' - 2$, where

$$\begin{aligned} \mathrm{E}[U_0(\widetilde{\mathbf{X}})] \;=\; &-\sum_{k=1}^{4} \alpha_k \sum_{n \in \mathcal{N}'_k} \Big( 2 \Pr[\widetilde{X}_n = 1] - 1 \Big) \\ &-\beta \sum_{n \in \mathcal{N}''} \Big( 4 \Pr[\widetilde{X}_n = 1, \widetilde{X}_{n+1} = 1] - 2 \Pr[\widetilde{X}_n = 1] - 2 \Pr[\widetilde{X}_{n+1} = 1] + 1 \Big), \quad (58) \end{aligned}$$

$$\mathrm{E}[g_1(X_{p-1}, X_{q+1})] = \sum_{x=0,1} \sum_{x'=0,1} g_1(x, x') \Pr[X_{p-1} = x, X_{q+1} = x'], \quad (59)$$

and

$$\mathrm{E}[g_2(X_{p'-1}, X_{q'+1})] = \sum_{x=0,1} \sum_{x'=0,1} g_2(x, x') \Pr[X_{p'-1} = x, X_{q'+1} = x']. \quad (60)$$

This implies that we can evaluate the NME $h(\widetilde{\mathbf{X}})$ using Supplementary Equation (57), provided that we know the probabilities associated with Supplementary Equations (58)-(60), which we can directly compute from the probabilities $\Pr[\mathbf{X} = \mathbf{x}]$ using the marginalization method discussed in Supplementary Section 4.

We can also apply the previous steps to more complex cases in which an allele is partitioned into an arbitrary number of subregions and arbitrary stretches of CpG sites. Due however to the complicated nature of the resulting formulas, we do not provide them here.

## 9 Computing the uncertainty coefficient

**Homozygous haplotypes.** Since we are considering diploid organisms, the probability of finding one of the two homologous alleles of a given haplotype in a biological sample can be taken to be equal to the probability of finding the other allele. We can therefore set $\Pr[A = 1] = \Pr[A = 2] = 1/2$, in which case Eqs. (8) and (11) in the main paper result in

$$\begin{aligned} I(\mathbf{X}; A) &= -\sum_{\mathbf{x}} \sum_{a=1,2} \Pr[\mathbf{X} = \mathbf{x}, A = a] \log_2 \frac{\Pr[\mathbf{X} = \mathbf{x}, A = a]}{\Pr[\mathbf{X} = \mathbf{x}] \Pr[A = a]} \\ &= Nh(\mathbf{X}) + \sum_{a=1,2} \Pr[A = a] \sum_{\mathbf{x}} \Pr[\mathbf{X} = \mathbf{x} \mid A = a] \log_2 \Pr[\mathbf{X} = \mathbf{x} \mid A = a] \\ &= Nh(\mathbf{X}) + \frac{1}{2} \sum_{a=1,2} \sum_{\mathbf{x}} \Pr[\mathbf{X} = \mathbf{x} \mid A = a] \log_2 \Pr[\mathbf{X} = \mathbf{x} \mid A = a] \\ &= N \left( h(\mathbf{X}) - \frac{1}{2} [h_1(\mathbf{X}) + h_2(\mathbf{X})] \right), \end{aligned} \quad (61)$$

with $h(\mathbf{X})$ being the NME of the methylation state $\mathbf{X}$ without knowing its allele of origin, and $h_1(\mathbf{X})$, $h_2(\mathbf{X})$ being the NMEs associated with each of the two homologous alleles. This result, together with Eq. (10) in the main paper, leads to

$$Q(\mathbf{X}; A) = 1 - \frac{1}{2} \left[ \frac{h_1(\mathbf{X}) + h_2(\mathbf{X})}{h(\mathbf{X})} \right], \quad (62)$$

which can be used to evaluate the uncertainty coefficient, given that we can compute the NMEs $h_1(\mathbf{X})$, $h_2(\mathbf{X})$, and $h(\mathbf{X})$.

Although we can compute the NMEs $h_1(\mathbf{X})$ and $h_2(\mathbf{X})$ using the methylation reads corresponding to each homologous allele and the method discussed in Supplementary Section 8, we cannot do so for the NME $h(\mathbf{X})$, since the probabilities of the allele-agnostic methylation state $\mathbf{X}$ within a haplotype do not necessarily follow a Gibbs distribution with potential energy of the form given by Supplementary Equation (15). However, one way to compute $h(\mathbf{X})$ is to realize that

$$\begin{aligned} \Pr[\mathbf{X} = \mathbf{x}] &= \Pr[\mathbf{X} = \mathbf{x} \mid A = 1] \Pr[A = 1] + \Pr[\mathbf{X} = \mathbf{x} \mid A = 2] \Pr[A = 2] \\ &= \frac{1}{2} \big( \Pr[\mathbf{X} = \mathbf{x} \mid A = 1] + \Pr[\mathbf{X} = \mathbf{x} \mid A = 2] \big) \\ &= \frac{1}{2} P(\mathbf{x}), \end{aligned} \quad (63)$$

where

$$P(\mathbf{x}) = \Pr[\mathbf{X} = \mathbf{x} \mid A = 1] + \Pr[\mathbf{X} = \mathbf{x} \mid A = 2]. \quad (64)$$

In this case,

$$
\begin{aligned}
h(\mathbf{X}) &= -\frac{1}{N}\sum_{\mathbf{x}} \Pr[\mathbf{X}=\mathbf{x}]\log_2 \Pr[\mathbf{X}=\mathbf{x}] \\
&= -\frac{1}{N}\sum_{\mathbf{x}} \left[\frac{1}{2}\,P(\mathbf{x})\right]\log_2\left[\frac{1}{2}\,P(\mathbf{x})\right] \\
&= \frac{1}{N}\left\{1-\frac{1}{2}\sum_{\mathbf{x}} P(\mathbf{x})\log_2 P(\mathbf{x})\right\}, \qquad (65)
\end{aligned}
$$

which allows computation of $h(\mathbf{X})$ by explicitly evaluating the required summation. Note that, for each state $\mathbf{x}$, we can compute the term $P(\mathbf{x})$ from the estimated CPEL models $\Pr[\mathbf{X}=\mathbf{x} \mid A=1]$ and $\Pr[\mathbf{X}=\mathbf{x} \mid A=2]$ using Supplementary Equation (64). However, we found that explicit evaluation of the summation in Supplementary Equation (65) is feasible only for haplotypes containing at most 16 CpG sites.

For haplotypes containing more than 16 CpG sites, we can compute an estimate $\widehat{h}(\mathbf{X})$ of $h(\mathbf{X})$ using Monte Carlo (MC) sampling. We can do so by setting

$$
\widehat{h}(\mathbf{X}) = \frac{1}{N}\left\{1-\frac{1}{L}\sum_{l=1}^{L}\log_2 P(\mathbf{x}_l)\right\}, \qquad (66)
$$

where $\{\mathbf{x}_1,\mathbf{x}_2,\ldots,\mathbf{x}_L\}$ are $L$ samples of the methylation state, with the $l$-th sample drawn from the conditional probabilities $\Pr[\mathbf{X}=\mathbf{x} \mid A=1]$ or $\Pr[\mathbf{X}=\mathbf{x} \mid A=2]$ with equal probability.

To draw a sample from the PMF $\Pr[\mathbf{X}=\mathbf{x} \mid A=1]$ (as well as from the PMF $\Pr[\mathbf{X}=\mathbf{x} \mid A=2]$), note that the CPEL model implies that the methylation state $\mathbf{X}$ within the $j$-th homologous allele is a first-order Markov chain with inhomogeneous transition probabilities. Indeed, as a direct consequence of Eqs. (1)-(3) in the main paper, we have that

$$
\begin{aligned}
&\Pr[X_n = x_n \mid X_{n-1}=x_{n-1},\ldots,X_1=x_1] \\
&\quad= \frac{\Pr[X_n=x_n, X_{n-1}=x_{n-1},\ldots,X_1=x_1]}{\sum_x \Pr[X_n=x, X_{n-1}=x_{n-1},\ldots,X_1=x_1]} \\
&\quad= \frac{g_n(x_n)\exp\left\{\alpha_n(2x_n-1)+\beta(2x_{n-1}-1)(2x_n-1)\right\}}{\sum_x g_n(x)\exp\left\{\alpha_n(2x-1)+\beta(2x_{n-1}-1)(2x-1)\right\}}, \qquad (67)
\end{aligned}
$$

where $g_n(x_n)$ is a term that takes into account the marginalization of the PMF $p(\mathbf{x})$ over CpG sites $n+1,n+2,\ldots,N$ (we set $g_N=1$, since there is no marginalization in this case), and $\alpha_n=\alpha_k$ if the $n$-th CpG site belongs to the $k$-th subregion of the allelic region $\mathcal{R}$. This

shows that the conditional probability of the methylation state at the $n$-th CpG site, given the methylation state at all upstream CpG sites $1, 2, \ldots, n-1$, depends only on the methylation state at CpG site $n-1$. As a consequence, $X_1, X_2, \ldots, X_N$ is a Markov chain with (inhomogeneous) first-order transition probabilities, given by

$$\Pr[X_n = x_n \mid X_{n-1} = x_{n-1}] = \frac{g_n(x_n) \exp\left\{\alpha_n(2x_n - 1) + \beta(2x_{n-1} - 1)(2x_n - 1)\right\}}{\sum_x g_n(x) \exp\left\{\alpha_n(2x - 1) + \beta(2x_{n-1} - 1)(2x - 1)\right\}}. \quad (68)$$

This Markov chain is initialized with a probability $\Pr[X_1 = x_1]$, given by

$$\Pr[X_1 = x_1] = \frac{g_1(x_1)}{Z} \exp\left\{\alpha_1(2x_1 - 1)\right\}, \quad (69)$$

which is obtained by marginalizing the PMF $\Pr[\mathbf{X} = \mathbf{x}]$ over CpG sites $2, 3, \ldots, N$.

As a consequence of the previous observation, we can recursively generate a sample $\mathbf{x}$ of the methylation state $\mathbf{X}$ by drawing a sample $x_1$ from the initial probability distribution $\Pr[X_1 = x_1]$, and by sequentially drawing samples $x_n$, $n = 2, 3, \ldots, N$, from the transition probability distributions $\Pr[X_n = x_n \mid X_{n-1} = x_{n-1}]$.

**Heterozygous haplotypes.** For a heterozygous haplotype with $M$ CpG sites $m = 1, 2, \ldots, M$ common to its homologous pair, we define the uncertainty coefficient by

$$Q(\widetilde{\mathbf{X}}; A) = \frac{1}{M} \frac{I(\widetilde{\mathbf{X}}; A)}{h(\widetilde{\mathbf{X}})}, \quad (70)$$

where $\widetilde{\mathbf{X}}$ is the vector of the methylation states at all common CpG sites, $I(\widetilde{\mathbf{X}}; A)$ is the mutual information between the methylation state at the common CpG sites and the allele of origin, and $h(\widetilde{\mathbf{X}})$ is the NME of $\widetilde{\mathbf{X}}$ regardless of the allele of origin. By following similar steps as in Supplementary Section 9 above, we can show that

$$Q(\widetilde{\mathbf{X}}; A) = 1 - \frac{1}{2} \left[ \frac{h_1(\widetilde{\mathbf{X}}) + h_2(\widetilde{\mathbf{X}})}{h(\widetilde{\mathbf{X}})} \right], \quad (71)$$

where $h_j(\widetilde{\mathbf{X}})$ is the NME of $\widetilde{\mathbf{X}}$ associated with the $j$-th homologous allele. We can use this expression to evaluate the uncertainty coefficient in a heterozygous haplotype, given that we can compute the NMEs $h_1(\widetilde{\mathbf{X}})$, $h_2(\widetilde{\mathbf{X}})$, and $h(\widetilde{\mathbf{X}})$, which we can do by the method discussed in Supplementary Section 8, for the NMEs $h_1(\widetilde{\mathbf{X}})$ and $h_2(\widetilde{\mathbf{X}})$, and by the method discussed in Supplementary Section 9, for the NME $h(\widetilde{\mathbf{X}})$ associated with the methylation vector $\widetilde{\mathbf{X}}$.

## 10 Hypothesis testing

Performing hypothesis testing, using one of the tests statistics $T$ given by Eqs. (4)-(6) in the main paper, requires that we know the cumulative distribution function $F_0(t; N) = \text{Pr}[T < t]$ of $T$ associated with a haplotype containing $N$ CpG sites, under the null hypothesis that the observed value of $T$ can be explained by the variability present in homozygous genomic regions. We can then calculate the $P$-value associated with an observation $t_*$ of $T$ by $p = 1 - F_0(t_*; N)$, which we can subsequently correct using the Benjamini-Hochberg procedure[21] while controlling for the false discover rate (FDR) to account for simultaneously testing a large number of hypotheses. We can then use the resulting $Q$-values to detect significant imbalances in mean methylation level and normalized methylation entropy between haplotype alleles, as well as to identify genetically informative haplotypes that demonstrate significant differences between the probability distributions of methylation within their homologous alleles.

Unfortunately, we do not know $F_0(t; N)$ and, therefore, we cannot compute $P$-values exactly. Nevertheless, we can compute an estimate

$$\widehat{F}_0(t; N) = \frac{1}{L} \sum_{l=1}^{L} I[t_l < t] \tag{72}$$

using an empirical bootstrap procedure, where $t_1, t_2, \ldots, t_L$ is a set of null statistics generated by the scheme described below and $I[\cdot]$ is the Iverson bracket, taking value 1 when its argument is true and 0 otherwise. This allows us to compute an estimate $\widehat{p}$ of the $P$-value associated with an observation $t_*$ of the test statistic $T$ by

$$\widehat{p} = 1 - \frac{1}{L+1} \sum_{l=1}^{L+1} I[t_l < t_*] = \frac{1}{L+1} \sum_{l=1}^{L+1} I[t_l \geq t_*] = \frac{1}{L+1} \left\{ 1 + \sum_{l=1}^{L} I[t_l \geq t_*] \right\}, \tag{73}$$

where we set $T_{L+1} = t_*$. It is however important to note that, for a haplotype that contains $N$ CpG sites, the statistical variability emanating from estimating the CPEL model is expected to increase with decreasing coverage and an increasing number of parameters that need to be estimated. To avoid complications arising from the fact that the coverage $C$ changes along the genome, and that the same is true for the number $K + 1$ of parameters to be estimated within each haplotype, we uniformly consider in our bootstrap procedure the coverage along the genome to be the minimum coverage $C_{\text{min}}$ observed within all analyzed haplotypes (we set $C_{\text{min}} = 5$), and assume that the same number $K_{\text{max}}(N) + 1$ of parameters must be estimated in each haplotype containing $N$ CpG sites, where $K_{\text{max}}(N)$ is the maximum $K$ value observed in all such haplotypes. This leads to a rather conservative approach to hypothesis testing that is

expected to result in a slight overestimation of the true $P$-values and lead to a robust test characterized by a reduced number of false positives. This follows from the fact that the generated null statistics in this case will contain the maximum amount of statistical variability present in the set of analyzed haplotypes.

With the previous facts in mind, we estimate $P$-values using the following scheme.

CPEL's hypothesis testing procedure

For a given $N \geq 1$:

1. Find all haplotypes containing $N$ CpG sites and calculate the maximum number $K_{\text{max}}(N) + 1$ of parameters that must be estimated within these haplotypes.
2. Find all genomic regions that have not been labeled as haplotypes and contain $N$ or more CpG sites.
3. Randomly select one of these regions with replacement and randomly choose a set of $N$ contiguous CpG sites within the selected region.
4. Randomly partition the set of WGBS reads overlapping these CpG sites into two complementary groups that contain the same number of reads (to avoid introducing read bias between the two homologous alleles) until each group results in a coverage of at least $C_{\text{min}}$ and, in each group, methylation is observed in at least $80\%$ of the CpG sites. If this cannot be done, repeat this step up to 20 times. If the step fails discard the region and go back to Step 3.
5. Keep randomly reducing the number of reads in each group, one read at a time, until the coverage in each group is between $C_{\text{min}}$ and $C_{\text{min}} + \Delta C$, for some small $\Delta C$ (we set $\Delta C = 2$) and methylation is observed in each group at least $80\%$ of the CpG sites. If this is not possible, then discard the region and go back to Step 3.
6. Perform parameter estimation of the CPEL models corresponding to the two homologous alleles using the WGBS reads obtained in Step 5. If parameter estimation is unsuccessful, return to Step 4. If parameter estimation is successful, compute the values of the test statistics $T_{\text{MML}}$, $T_{\text{NME}}$, and $T_{\text{PDM}}$ under the null hypothesis, swap a pair of reads in the two groups at random and redo parameter estimation. Repeat Step 6 a maximum of 10 times for each region obtained in Step 3.
7. Repeat Steps 3-6 until a required minimum number $L$ (we take $L \geq$ 1,000) of null statistic values are obtained.

8. For an observed test statistic value $t_*$ that is computed from a haplotype containing $N$ CpG sites, estimate the associated $P$-value using Supplementary Equation (73), where $t_1, t_2, \ldots, t_L$ are the values of the corresponding test statistic under the null hypothesis.

Note that larger values of $\Delta C$ in Step 5 will lead to more detections in the hypothesis testing step at the expense of a higher number of false positives. This follows from the fact that, in this case, some null statistics will be computed at coverages that are much larger than $C_{\text{min}}$, whereas some test statistics will be computed at coverages close to $C_{\text{min}}$. Finally, generating at least 1,000 null statistics for each $N$ is sufficient for consistent hypothesis testing at a significance level of $5\%$. That is, if an observed test statistic $t_*$ is truly significant at a $5\%$ significance level, then the estimate $\widehat{p}$ of the true $P$-value computed in Step 8 using at least 1,000 null statistics will generally deem $t_*$ to be significant or near significant[22].

# Supplementary Discussion

In a previous ASM study[1], Gertz et al. performed ASM analysis using reduced representation bisulfite sequencing (RRBS) data[23]. We identified two main methodological flaws in their work. First, alignment was performed by transforming all cytosines (Cs) in the RRBS reads, and the reference genome into thymidines (Ts) and by using an in-house script to align these reads to a C-to-T transformed reference genome using Bowtie[24]. However, this is not a good alignment strategy (a more appropriate bisulfite sequencing alignment technique should have been used, such as Bismark[25]), since it can result in a significant number of methylation reads not being aligned to the reference genome, thus seriously affecting subsequent analyses. Second, each SNP was associated with (possibly) multiple CpG sites and allele-specific analysis of mean methylation was performed at these CpG sites using the NPI method and Fisher's exact test. As a result, marginal statistical analysis was performed at each individual SNP, which cannot capture correlations between the methylation states at neighboring CpG sites and ignores the joint effects of multiple genetic differences on methylation stochasticity. As has been shown by Jenkinson et al.[9], a marginal approach to detecting methylation imbalances may also lead to loss of specificity (true negative rate) and sensitivity (true positive rate), which will seriously affect its statistical performance. In addition, such an approach may lead to sensitivity that coincides with the Type I error rate (false positive rate), indicating a performance that is no better than random guessing. Finally, independently applying Fisher's exact test can lead to poor detection performance that is characterized by unacceptably low sensitivity and specificity. Due to these shortcomings, we believe that the biological conclusions reached by Gertz et al.[1] cannot be trusted.

We also found several methodological issues with the recent work of Onuchic et al.[7], which can directly affect the validity of the conclusions reached in that study. These investigators performed ASM analysis at groups of $4$ CpG sites (known as epialleles), with each group being associated with an individual SNP, by employing WGBS data and by modeling methylation stochasticity using the NPD method. Individual SNPs were then identified for which significant imbalances in mean methylation levels were detected between the associated homologous epialleles using Fisher's exact test on the percentage of methylated and unmethylated CpG sites observed within the two epialleles. Methylation stochasticity was also evaluated using Shannon's entropy and the relationship between genetic variation at individual SNPs and epiallelic stochasticity was quantified using the uncertainty coefficient (previously was referred to by Onuchic et al. as the coefficient of constraint).

Although, the Onuchic et al. approach to ASM analysis takes into account correlations between the methylation states of neighboring CpG sites, this is limited to correlations between the methylation states at CpG sites of individual epialleles. Moreover, and similarly to the approach of Gertz et al.[1], this approach ignores the joint effects of multiple genetic differences on methylation stochasticity. In addition, and due to the fact that current WGBS technology cannot produce data with sufficient coverage, using the NPD method for ASM analysis may not be appropriate since, as we discuss in in this paper, sound model estimation is highly problematic in this case. Notably, the data used by Onuchic et al. are characterized by coverages ranging from $4\times$ to about $75\times$, which are definitely not adequate for reliably estimating the required $16$ epiallelic probabilities. This problem is further exacerbated by the fact that estimation of epiallelic probabilities requires WGBS reads for which the methylation state is observed at all $4$ CpG sites, which effectively reduces available coverage. Inaccurate estimation of epiallelic probabilities obtained by using insufficient data, can result in erroneous computations of methylation entropies and uncertainty coefficients[6], which can seriously affect downstream ASM analysis and limit the reliability of this analysis to a small number of SNPs exhibiting sufficient WGBS coverage. We should note here that, in an effort to address these issues, Onuchic et al. pooled WGBS reads across all tissues and donors used in their study achieving a 1,691$\times$ total coverage. However, pooling multi-tissue/donor data is biologically questionable, since it can seriously obscure tissue- and donor-specific information and can therefore result in highly questionable and misleading biological conclusions.

In addition to the above, the hypothesis testing methods employed by the previous two studies are limited to identifying only significant mean methylation imbalances, and cannot be used to detect imbalances in methylation entropies or identify genetically informative haplotypes exhibiting significant differences between the probability distributions of methylation within their homologous alleles, which is especially important for providing a more complete picture of methylation stochasticity in ASM studies. Most importantly however these methods do not take into account variability present in homozygous genomic regions and, therefore, cannot test true allele-specific methylation events, an issue that can seriously affect their sensitivity (true positive rate) and specificity (true negative rate). We should finally note that the methodological issues associated with the work of Onuchic et al. become more pronounced when the objective is haplotype-dependent allele-specific methylation analysis, since haplotypes may contain more than $4$ CpG sites (see Supplementary Figure 4b). As a consequence, we cannot recommend their approach for use in genome-wide hap-ASM analysis.

# Supplementary Figures

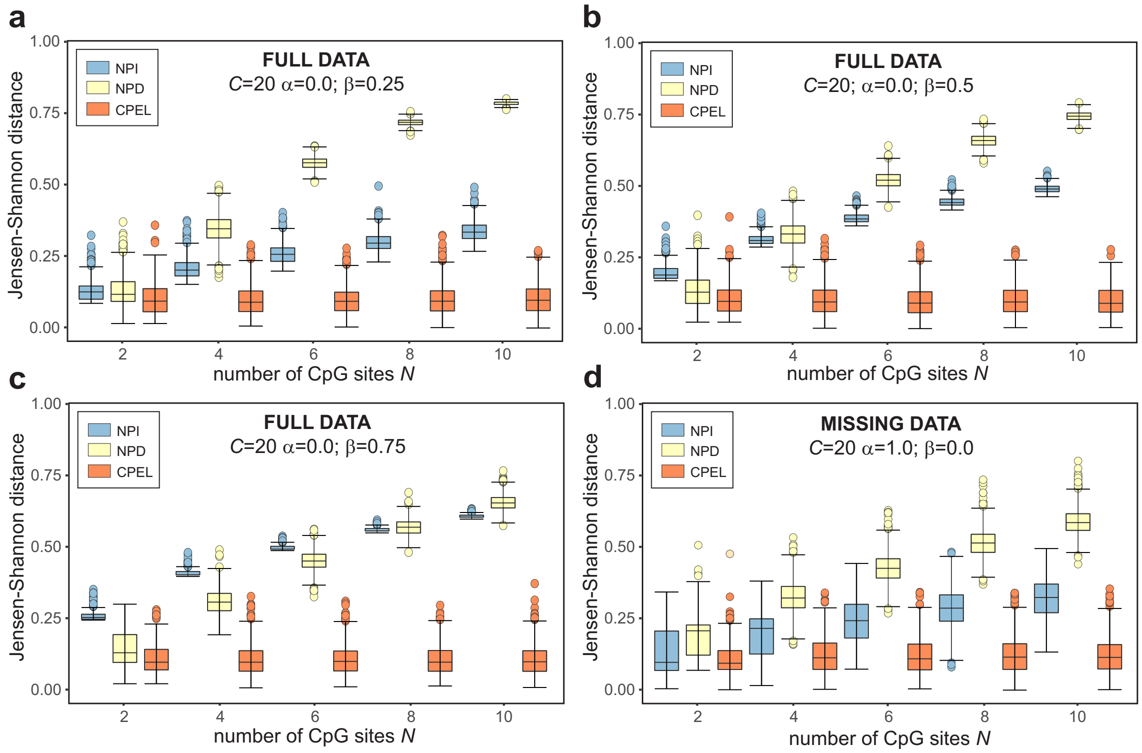


**Supplementary Figure 1 Additional model estimation results using simulated data.** Boxplots depicting distributions of Jensen-Shannon distance values when comparing estimated probability distributions of methylation (PDMs) to the true PDMs for a wide range of conditions using the NPI, NPD, and CPEL methods and simulated data. Estimation was independently performed 1,000 times, each using 20 fully observed or 20 partially observed reads, as indicated. The results demonstrate a consistently superior performance of the CPEL method for correctly estimating methylation probabilities in a haplotype allele when compared to the NPI and NPD methods. **a-c** Fully observed correlated data and increased number of CpG sites. **d** Partially observed non-correlated data and increased number of CpG sites. Center line of box: median value; box bounds: 25th and 75th percentiles; lower whisker: larger of minimum value and 25th percentile minus 1.5 $\times$ interquartile range; upper whisker: smaller of maximum value and 75th percentile plus 1.5 $\times$ interquartile range.

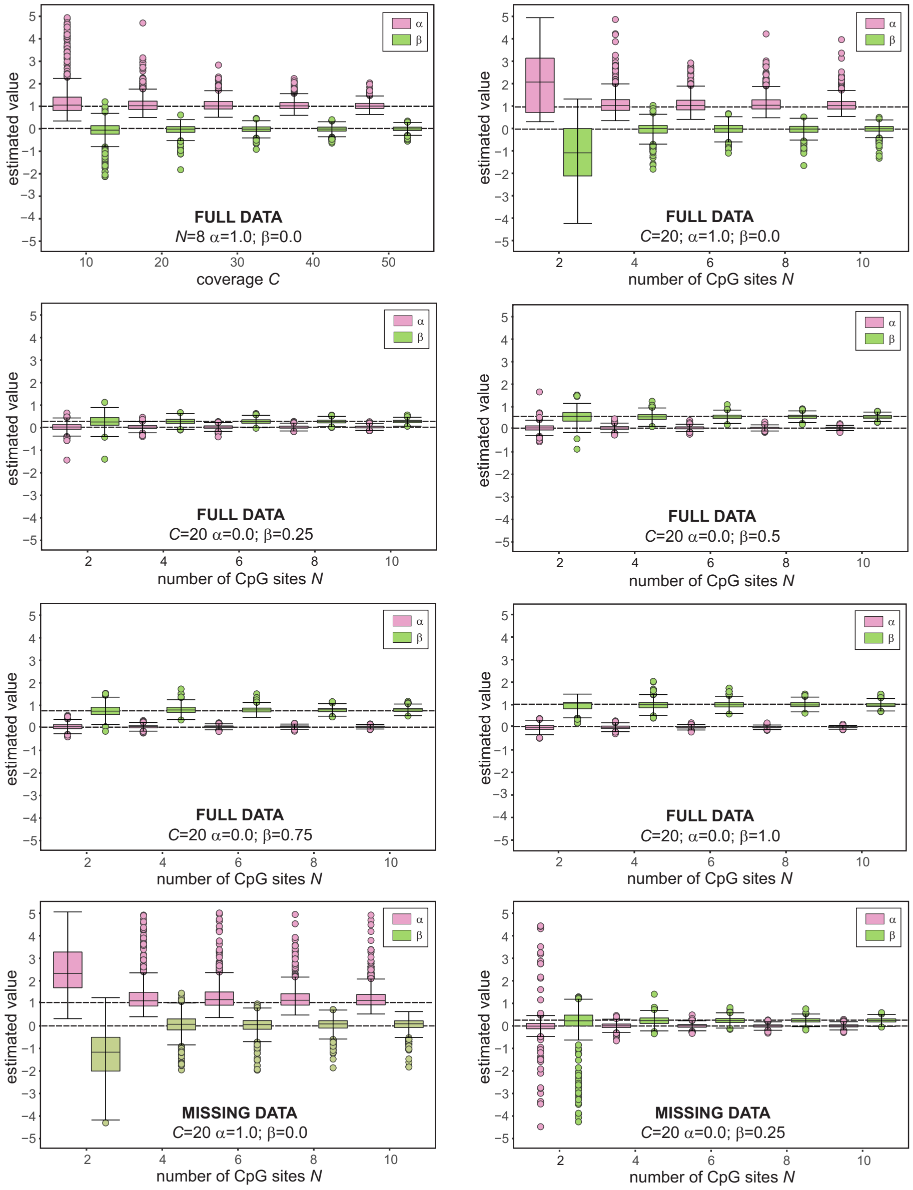


**Supplementary Figure 2 Simulated evaluation of parameter estimation performance.** Performance evaluation examples of estimating the parameters of the CPEL model using simulated data corresponding to the examples depicted in Fig. 3 of the main paper and in Supplementary Figure 1. Estimation was independently performed 1,000 times, each using 20 fully observed or 20 partially observed reads, as indicated. The boxplots depict distributions of estimated parameter values associated with the methylation potential energy landscape, whereas the dashed lines depict the true parameter values. Center line of box: median value; box bounds: 25th and 75th percentiles; lower whisker: larger of minimum value and 25th percentile minus 1.5 $\times$ interquartile range; upper whisker: smaller of maximum value and 75th percentile plus 1.5 $\times$ interquartile range.

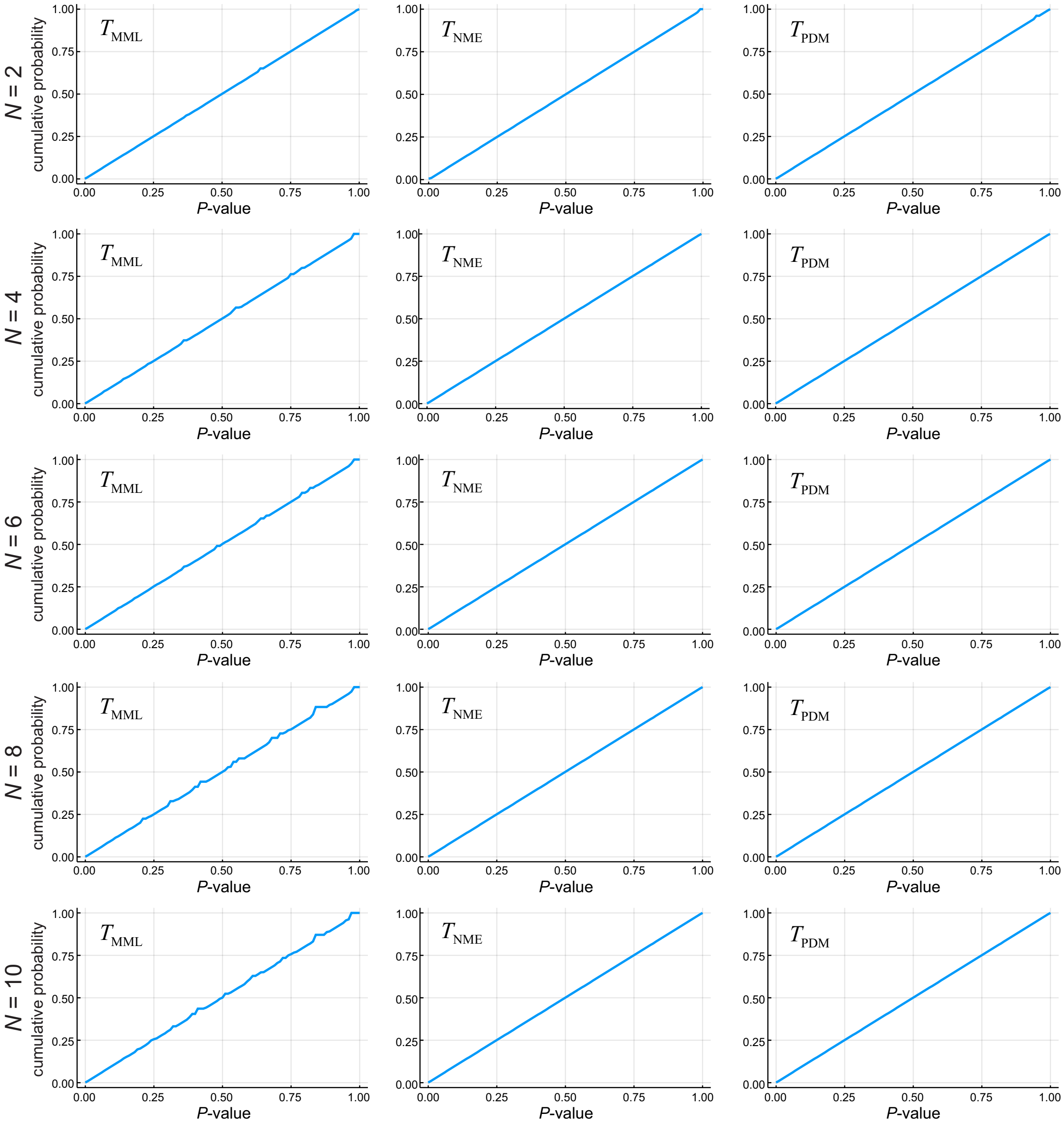


**Supplementary Figure 3 Estimated cumulative distributions of 'null' *P*-values using simulations.** Representative examples of empirically estimated cumulative distribution functions of $P$-values obtained by CPEL's one-sided empirical bootstrap hypothesis testing procedure under the null hypothesis using simulations. The test statistics given by Eqs. (4)-(6) in the main paper lead to almost linear cumulative distribution functions for haplotypes containing $N = 2, 4, 6, 8, 10$ CpG sites, demonstrating that the probability distributions of the $P$-values are close to being uniform under the null hypothesis. In these simulations, $K_{\text{max}}(2) = 2$, $K_{\text{max}}(4) = 3$, and $K_{\text{max}}(6) = K_{\text{max}}(8) = K_{\text{max}}(10) = 4$, in agreement with the values observed in all haplotypes analyzed by the CPEL method in the real data.

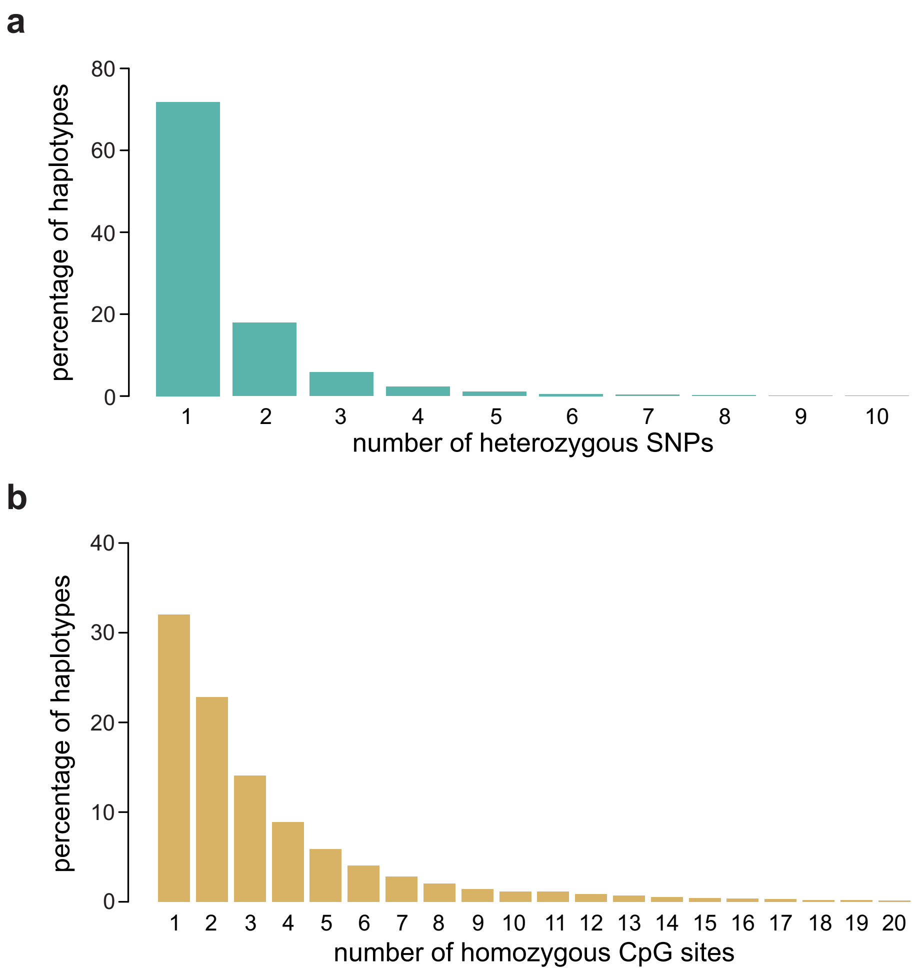


**Supplementary Figure 4 Haplotype distributions in the real data.** Distributions of 715,155 haplotypes identified by read-based SNP phasing. **a** Distribution in terms of the number of heterozygous SNPs found within the haplotypes. For clarity, this distribution is limited to 10 SNPs, although haplotypes were found to be associated with up to 121 SNPs. **b** Distribution in terms of the number of homozygous CpG sites found within the haplotypes.

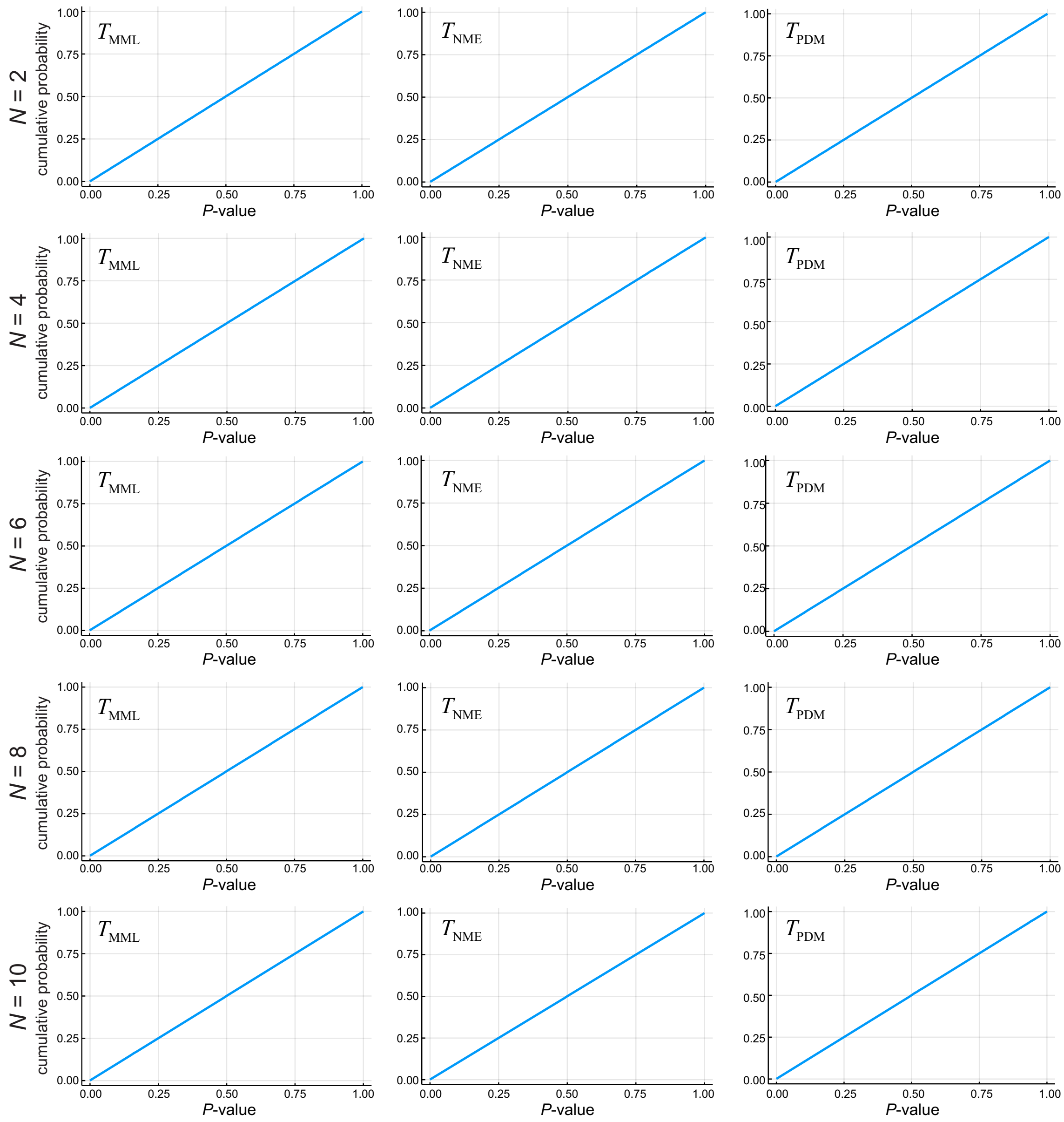


**Supplementary Figure 5 Estimated cumulative distributions of 'null' *P*-values using the real data.** Examples of empirically estimated cumulative distribution functions of $P$-values obtained by CPEL's one-sided empirical bootstrap hypothesis testing procedure under the null hypothesis using the real data corresponding to the aorta tissue. The test statistics given by Eqs. (4)-(6) in the main paper lead to linear cumulative distribution functions for haplotypes containing $N = 2, 4, 6, 8, 10$ CpG sites, demonstrating that the probability distributions of the $P$-values are uniform. These results are representative of the general behavior in the data.

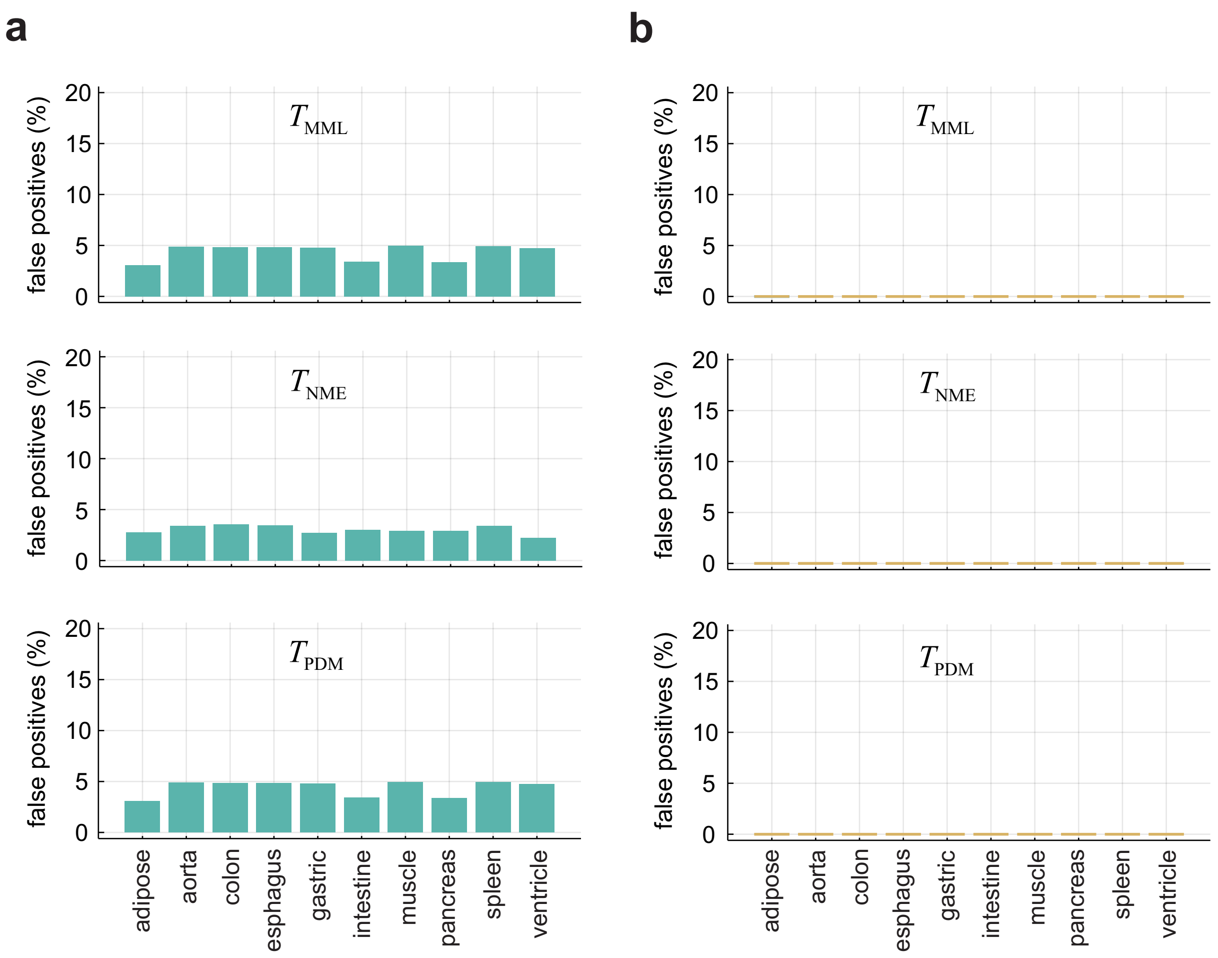


**Supplementary Figure 6 Performance of hypothesis testing in the real data.** **a** Percentage of false positives produced by CPEL's one-sided empirical bootstrap procedure for hypothesis testing, using the three test statistics given by Eqs. (4)-(6) in the main paper, when applied on 'null' genomic regions which are not labeled to be haplotypes in the real data. As expected, no more than $5\%$ of these regions exhibit significant methylation imbalances under the null hypothesis ($P$-value $\leq 0.05$). **b** Controlling the false discovery rate (FDR) using the Benjamini-Hochberg procedure with a $0.05$ threshold for the adjusted $P$-values produces no false positives in all cases. Number of 'null' genomic regions considered: 34,095 (adipose); 45,666 (aorta); 49,017 (colon); 51,150 (esophagus); 34,857 (gastric); 40,803 (intestine); 37,029 (muscle); 47,607 (pancreas); 50,493 (spleen); 33,435 (ventricle).

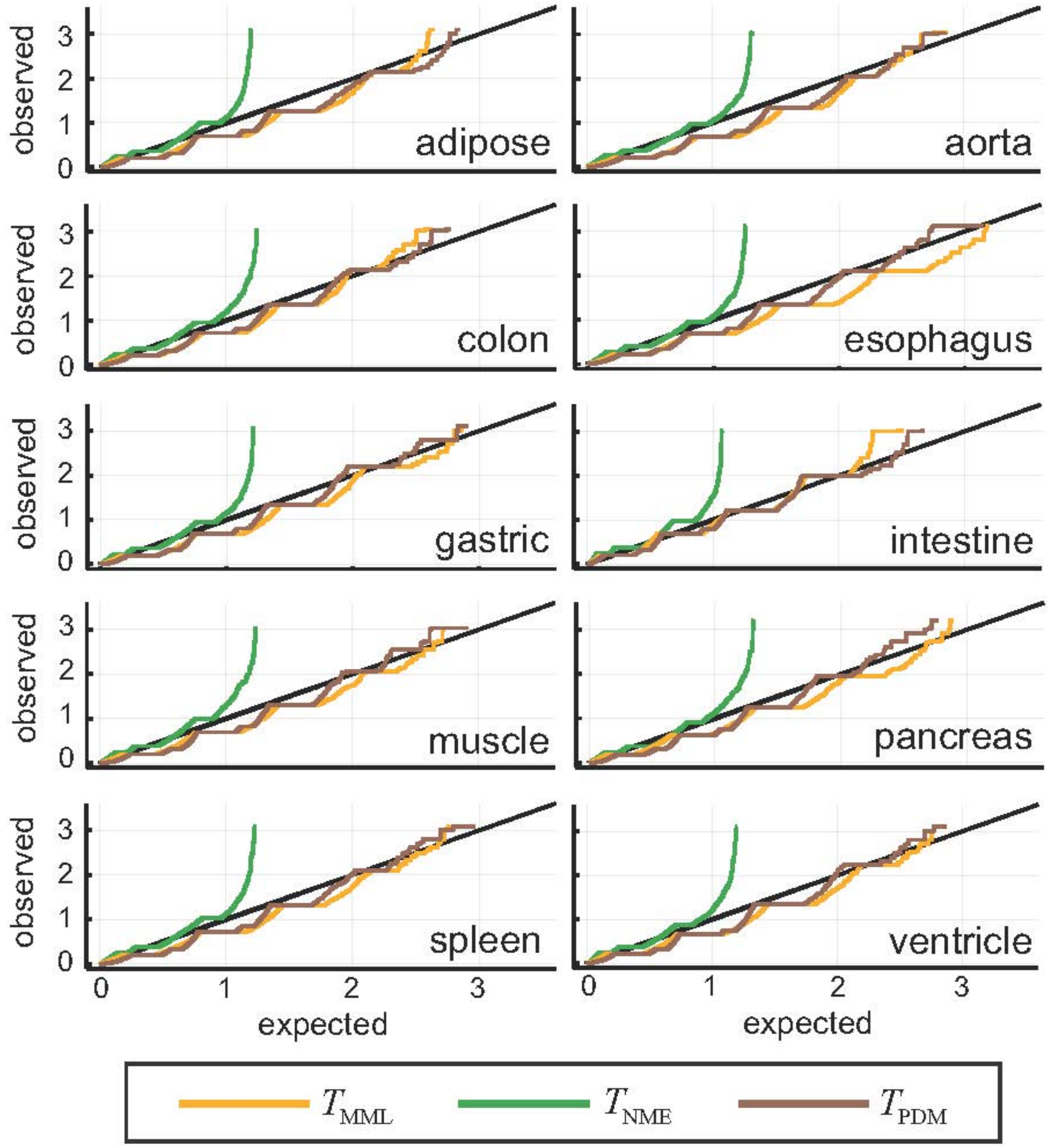


**Supplementary Figure 7 Q-Q plot comparisons of *P*-values in the real data.** Q-Q plots, for each test statistic given by Eq. (5) in the main paper and each tissue of the real data, of quantiles of $-\log_{10}$ $P$-values observed within haplotypes versus expected quantiles of $-\log_{10}$ $P$-values under the null hypothesis. Although some small $-\log_{10}$ $P$-values are located on the diagonal line in each plot, other small values are placed slightly below this line. This is due to the conservative nature of CPEL's hypothesis testing procedure, which results in slight overestimation of the true $P$-values.

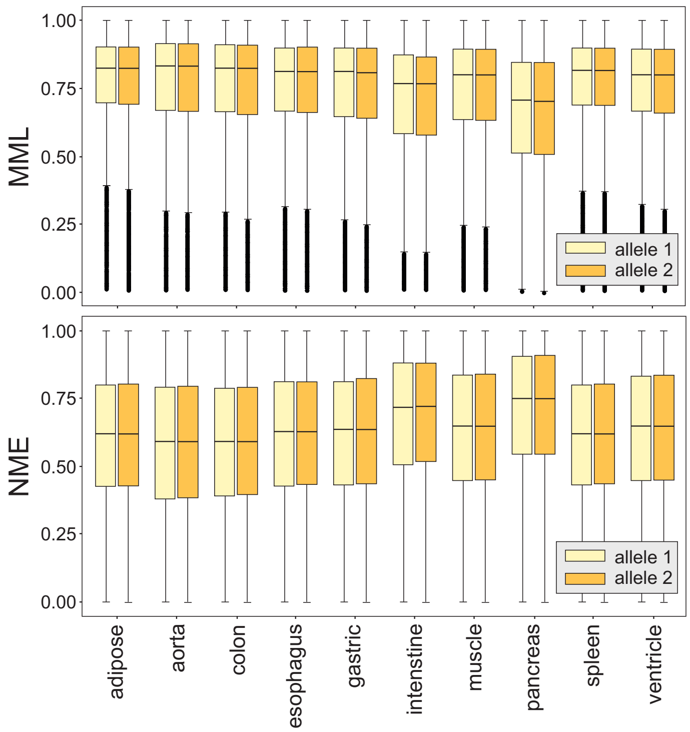


**Supplementary Figure 8 Distributions of MML and NME values within haplotypes in the real data.** Boxplots of distributions of MML and NME values associated with the homologous alleles of the haplotypes identified by the CPEL method in each tissue of the real data. Number of haplotypes considered: 140,358 (adipose); 210,584 (aorta); 160,605 (colon); 162,780 (esophagus); 143,100 (gastric); 45,162 (intestine); 163,066 (muscle); 179,152 (pancreas); 114,089 (spleen); 148,478 (ventricle). Center line of box: median value; box bounds: 25th and 75th percentiles; lower whisker: larger of minimum value and 25th percentile minus 1.5 $\times$ interquartile range; upper whisker: smaller of maximum value and 75th percentile plus 1.5 $\times$ interquartile range.

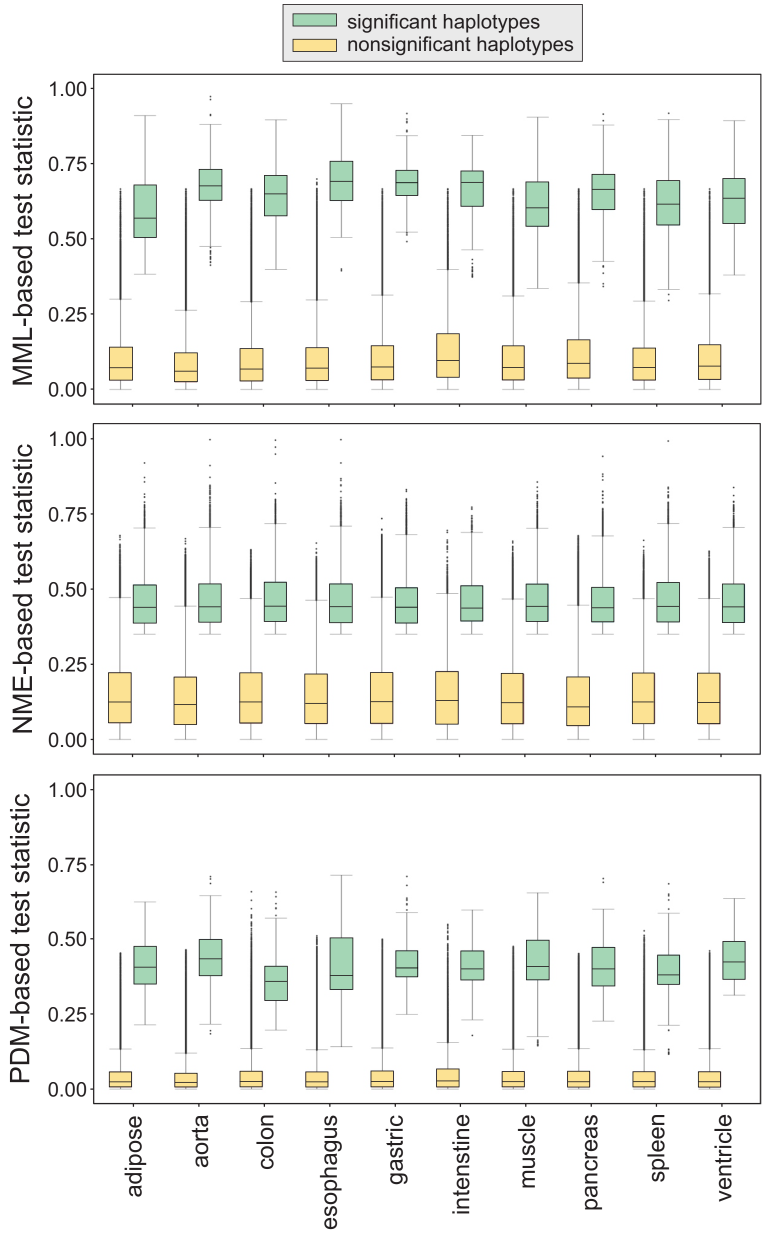


**Supplementary Figure 9 Distributions of test statistic values within haplotypes in the real data.** Boxplots of distributions of test statistic values associated with significant and nonsignificant haplotypes identified by the CPEL method in each tissue of the real data. Overlaps between significant and non-significant test statistic values are due to the fact that the critical value at which the null hypothesis is rejected depends on the number of CpG sites contained in a haplotype. Number of haplotypes considered: 135,259 (adipose); 204,506 (aorta); 157,090 (colon); 159,186 (esophagus); 137,582 (gastric); 44,440 (intestine); 157,864 (muscle); 172,884 (pancreas); 155,323 (spleen); 135,833 (ventricle).

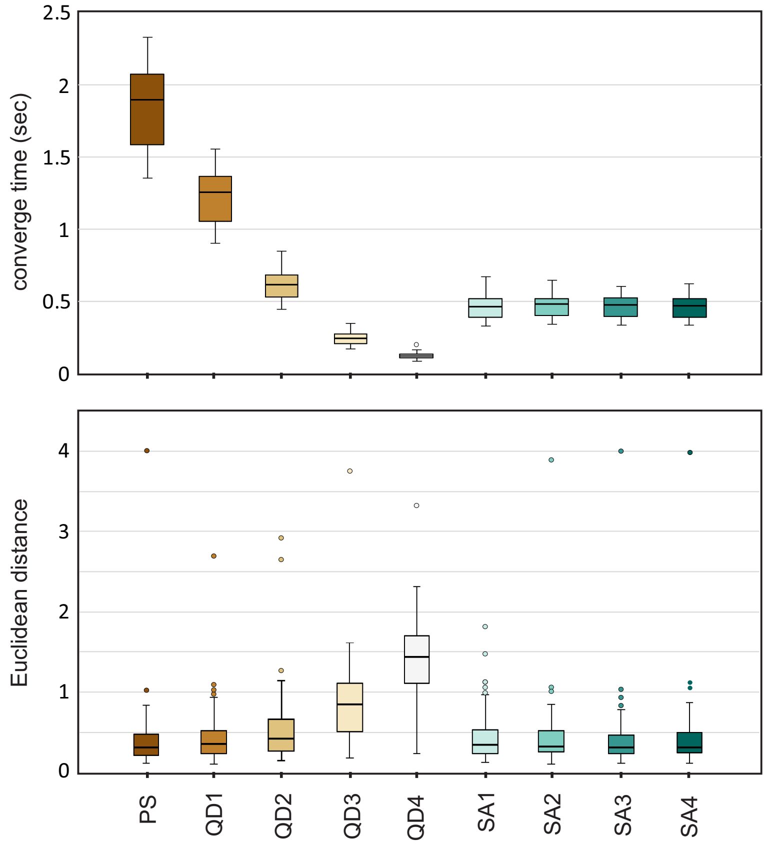


**Supplementary Figure 10 Evaluation of optimization algorithms for parameter estimation.** Boxplots of convergence times and Euclidean distances between estimated and true parameter values of a CPEL model computed by three global optimization algorithms: Particle Swarm (PS), QuadDIRECT (QD), and Simulated Annealing (SA). These boxplots were generated for Case 5 in Supplementary Table 8 by performing parameter estimation 100 times using different initializations. This case is the most challenging among the five considered, since it involves missing data at the two extremes (beginning and end) and in the middle of the allele. Similar results were obtained for the other four cases in Supplementary Table 8. The maximum number of function evaluations in QuadDIRECT was taken to be 2500 (QD1), 1250 (QD2), 500 (QD3), and 250 (QD4). The temperature reduction factor in SA was taken to be $10^{-2}$ (SA1), $10^{-3}$ (SA2), $10^{-4}$ (SA3), and $10^{-5}$ (SA4). Center line of box: median value; box bounds: 25th and 75th percentiles; lower whisker: larger of minimum value and 25th percentile minus 1.5 $\times$ interquartile range; upper whisker: smaller of maximum value and 75th percentile plus 1.5 $\times$ interquartile range.

# Supplementary Tables

**Supplementary Table 1 Data-set used for hap-ASM analysis.**

| Name | Tissue Type | Donor ID | GWC | NHC | A1C | A2C |
|---|---|---|---|---|---|---|
| adipose | Adipose tissue | STL003 | 39 | 42 | 8 | 8 |
| aorta | Aorta | STL003 | 64 | 69 | 13 | 13 |
| colon | Sigmoid colon | STL003 | 48 | 53 | 10 | 10 |
| esophagus | Esophagus | STL003 | 49 | 50 | 10 | 10 |
| gastric | Gastric | STL003 | 40 | 43 | 8 | 8 |
| intestine | Small intestine | STL003 | 18 | 20 | 5 | 5 |
| muscle | Psoas muscle | STL003 | 44 | 47 | 9 | 9 |
| pancreas | Pancreas | STL003 | 48 | 49 | 10 | 10 |
| spleen | Spleen | STL003 | 45 | 49 | 10 | 10 |
| ventricle | Right ventricle | STL003 | 41 | 44 | 9 | 9 |

GWC: average genome-wide WGBS coverage; NHC: average genome-wide WGBS coverage within non-haplotype regions; A1C: average WGBS coverage within haplotype alleles of the first genetic origin; A2C: average WGBS coverage within haplotype alleles of the second genetic origin.

**Supplementary Table 2 Odds-ratio analysis of MML-haps overlapping genomic features/regions.**

| **Features/Regions** | **OR** | **95% CI** | ***P*-value** |
|---|---|---|---|
| CGIs | 2.50 | $[1.81, 3.35]$ | $< 0.001$ |
| CGI shores | 1.47 | $[1.26, 1.69]$ | $< 0.001$ |
| CGI shelves | 0.83 | $[0.66, 1.03]$ | 0.09 |
| open seas | 0.79 | $[0.70, 0.90]$ | $< 0.001$ |
| promoters | 1.67 | $[1.34, 2.06]$ | $< 0.001$ |
| exons | 1.57 | $[1.29, 1.88]$ | $< 0.001$ |
| introns | 1.11 | $[1.02, 1.20]$ | 0.01 |
| intergenic | 0.96 | $[0.88, 1.04]$ | 0.31 |
| enhancers | 1.86 | $[1.67, 2.07]$ | $< 0.001$ |
| CG-rich | 1.57 | $[1.36, 1.81]$ | $< 0.001$ |
| CG-poor | 0.64 | $[0.56, 0.74]$ | $< 0.001$ |
| transcriptional | 1.82 | $[1.66, 2.00]$ | $< 0.001$ |
| non-transcriptional | 0.56 | $[0.51, 0.63]$ | $< 0.001$ |

Enrichment analysis of MML-haps, identified by CPEL, overlapping selected genomic fetures/regions using the odds-ratio (OR) statistic and Fisher's two-sided exact test. OR $> 1$ indicates enrichment; OR $< 1$ indicates depletion; CI: confidence interval.

**Supplementary Table 3 Odds-ratio analysis of NME-haps overlapping genomic features/regions.**

| Genomic Features | OR | 95% CI | *P*-value |
|---|---|---|---|
| CGIs | 0.10 | $[0.08, 0.12]$ | $< 0.001$ |
| CGI shores | 0.47 | $[0.45, 0.48]$ | $< 0.001$ |
| CGI shelves | 0.72 | $[0.69, 0.75]$ | $< 0.001$ |
| open seas | 1.76 | $[1.72, 1.81]$ | $< 0.001$ |
| promoters | 0.63 | $[0.59, 0.66]$ | $< 0.001$ |
| exons | 0.43 | $[0.41, 0.46]$ | $< 0.001$ |
| introns | 0.88 | $[0.87, 0.89]$ | $< 0.001$ |
| intergenic | 1.15 | $[1.14, 1.17]$ | $< 0.001$ |
| enhancers | 0.64 | $[0.62, 0.65]$ | $< 0.001$ |
| CG-rich | 0.45 | $[0.43, 0.47]$ | $< 0.001$ |
| CG-poor | 2.15 | $[2.06, 2.23]$ | $< 0.001$ |
| transcriptional | 0.58 | $[0.57, 0.60]$ | $< 0.001$ |
| non-transcriptional | 1.23 | $[1.20, 1.26]$ | $< 0.001$ |

Enrichment analysis of NME-haps, identified by CPEL, overlapping selected genomic fetures/regions using the odds-ratio (OR) statistic and Fisher's two-sided exact test. OR $> 1$ indicates enrichment; OR $< 1$ indicates depletion; CI: confidence interval.

**Supplementary Table 4 Odds-ratio analysis of PDM-haps overlapping genomic features/regions.**

| Genomic Features | OR | 95% CI | *P*-value |
|---|---|---|---|
| CGIs | 0.46 | $[0.18, 0.94]$ | 0.03 |
| CGI shores | 0.64 | $[0.50, 0.80]$ | $< 0.001$ |
| CGI shelves | 0.80 | $[0.62, 1.01]$ | 0.07 |
| open seas | 1.48 | $[1.24, 1.77]$ | $< 0.001$ |
| promoters | 1.01 | $[0.74, 1.34]$ | 0.94 |
| exons | 0.90 | $[0.68, 1.17]$ | 0.49 |
| introns | 0.91 | $[0.83, 1.00]$ | 0.05 |
| intergenic | 1.14 | $[1.04, 1.25]$ | 0.01 |
| enhancers | 1.42 | $[1.25, 1.61]$ | $< 0.001$ |
| CG-rich | 0.65 | $[0.52, 0.81]$ | $< 0.001$ |
| CG-poor | 1.59 | $[1.26, 2.03]$ | $< 0.001$ |
| transcriptional | 1.27 | $[1.13, 1.42]$ | $< 0.001$ |
| non-transcriptional | 1.13 | $[0.97, 1.31]$ | 0.12 |

Enrichment analysis of PDM-haps, identified by CPEL, overlapping selected genomic fetures/regions using the odds-ratio (OR) statistic and Fisher's two-sided exact test. OR $> 1$ indicates enrichment; OR $< 1$ indicates depletion; CI: confidence interval.

**Supplementary Table 5 Odds-ratio analysis of significant haplotypes overlapping imprinted genes.**

| Haplotypes | OR | 95% CI | *P*-value |
|---|---|---|---|
| MML-haps | 74.73 | $[42.58, 126.99]$ | $< 0.001$ |
| NME-haps | 1.18 | $[0.49, 2.38]$ | $0.56$ |
| PDM-haps | 25.58 | $[7.74, 64.66]$ | $< 0.001$ |

Enrichment analysis of statistically significant haplotypes overlapping the promoter regions of 107 known imprinted genes, obtained from http://www.geneimprint.com, using the odds-ratio (OR) statistic and Fisher's two-sided exact test. OR $> 1$ indicates enrichment; OR $< 1$ indicates depletion; CI: confidence interval.

**Supplementary Table 6 Genes with promoters overlapping MML-haps.**

| Gene | Imprinted | Gene | Imprinted |
|---|---|---|---|
| *AGPAT4* | | *MGAT2* | |
| *BCLAF1* | | *MIR4684* | |
| *C22orf31* | | *MTRR* | |
| *C3* | | *NAV1* | |
| *C8G* | | *NHSL1* | |
| *CD180* | | *NNAT* | * |
| *CEP112* | | *NOSTRIN* | |
| *CRISP2* | | *NT5DC3* | |
| *DEFA11P* | | *OPRM1* | |
| *FAM222A* | | *OTOP1* | |
| *FAM240A* | | *PTBP2* | |
| *FES* | | *PYROXD2* | |
| *FMN1* | | *RFPL3* | |
| *GDPD1* | | *RPLP0P2* | |
| *GNAS* | * | *RTN4IP1* | |
| *GNB4* | | *S100A14* | |
| *GSDMD* | | *SCGB1D2* | |
| *GZMM* | | *SCRN2* | |
| *H19* | * | *SNORD114-6* | |
| *IQSEC1* | | *SNURF* | * |
| *KRTAP2-4* | | *SPATC1L* | |
| *LINC00298* | | *SYT3* | |
| *LINC01168* | | *TMEM169* | |
| *LRP10* | | *VTRNA2-1* | * |
| *MAGEL2* | * | *ZNF205-AS1* | |
| *MEF2D* | | *ZNF79* | |
| *MESTIT1* | * | | |

Genes with promoter regions overlapping haplotypes identified by the CPEL method to exhibit significant imbalances in mean methylation levels (MML-haps) in at least one tissue in the real data. The marked genes are known to be imprinted.

**Supplementary Table 7 Significant haplotypes and imprinted genes**.

| Imprinted gene | MML-hap | NME-hap | PDM-hap | # |
|---|---|---|---|---|
| *GNAS* | * | | | 6 |
| | * | * | | 0 |
| | * | | * | 0 |
| | * | * | * | 0 |
| *H19* | * | | | 1 |
| | * | * | | 0 |
| | * | | * | 2 |
| | * | * | * | 0 |
| *MAGEL2* | * | | | 3 |
| | * | * | | 1 |
| | * | | * | 2 |
| | * | * | * | 0 |
| *MESTIT1* | * | | | 1 |
| | * | * | | 0 |
| | * | | * | 0 |
| | * | * | * | 0 |
| *NNAT* | * | | | 0 |
| | * | * | | 1 |
| | * | | * | 0 |
| | * | * | * | 0 |
| *SNURF* | * | | | 1 |
| | * | * | | 0 |
| | * | | * | 0 |
| | * | * | * | 0 |
| *VTRNA2-1* | * | | | 3 |
| | * | * | | 0 |
| | * | | * | 1 |
| | * | * | * | 0 |

Number of haplotypes with a particular ASM significance pattern, identified by the CPEL method across all tissues in the real data, which overlap the promoter regions of the imprinted genes in Supplementary Table 6. Although the CPEL method identified 32 haplotypes, only 22 exhibited significant imbalances in mean methylation level in the data.

**Supplementary Table 8 Cases for comparing optimization algorithms.**

| Case | 1 | 2 | 3 | 4 | 5 |
|---|---|---|---|---|---|
| $M$ | 20 | 20 | 40 | 40 | 60 |
| $K$ | 1 | 3 | 3 | 6 | 6 |
| $\{N_k\}$ | $\{9\}$ | $\{3,3,3\}$ | $\{3,3,3\}$ | $\{3,3,3,3,3,3\}$ | $\{3,3,3,3,3,3\}$ |
| $\{\alpha_k\}$ | $\{1\}$ | $\{1,0,-1\}$ | $\{1,0,-1\}$ | $\{1,0,-1,0,1,0\}$ | $\{1,0,-1,0,1,0\}$ |
| $\beta$ | $0.5$ | $0.5$ | $0.5$ | $0.5$ | $0.5$ |
| MDE | NO | NO | YES | YES | YES |
| MDM | NO | NO | NO | NO | YES |

$M$: number of methylation reads within an allele; $K$: number of subregions within the allele; $\{N_k\}$: numbers of CpG sites within the allele; $\{\alpha_k\}$, $\beta$: true parameter values; MDE: missing data at the two extremes (beginning and end) of the allele; MDM: missing data in the middle of the allele.

# Supplementary References


1. Gertz, J. et al. Analysis of DNA methylation in a three-generation family reveals widespread genetic influence on epigenetic regulation. *PLoS Genet.* **7**, e1002228 (2011).
2. Fang, F. et al. Genomic landscape of human allele-specific DNA methylation. *Proc. Natl. Acad. Sci. USA* **109**, 7332–7337 (2012).
3. Hermann, A., Goyal, R. & Jeltsch, A. The Dnmt1 DNA-(cytosine-C5)-methyltransferase methylates DNA processively with high preference for hemimethylated target sites. *J. Biol. Chem.* **279**, 48350–48359 (2004).
4. Vilkaitis, G., Suetake, I., Klimasauskas, S. & Tajima, S. Processive methylation of hemimethylated CpG sites by mouse Dnmt1 DNA methyltransferase. *J. Biol. Chem.* **280**, 64–72 (2005).
5. Jeltsch, A. On the enzymatic properties of Dnmt1: specificity, processivity, mechanism of linear diffusion and allosteric regulation of the enzyme. *Epigenetics* **1**, 63–66 (2006).
6. Jenkinson, G., Pujadas, E., Goutsias, J. & Feinberg, A. P. Potential energy landscapes identify the information-theoretic nature of the epigenome. *Nat. Genetics* **49**, 719–729 (2017).
7. Onuchic, V. et al. Allele-specific epigenome maps reveal sequence-dependent stochastic switching at regulatory loci. *Science* **361**, eaar3146 (2018).
8. Pressé, S., Ghosh, K., Lee, J. & Dill, K. A. Principles of maximum entropy and maximum caliber in statistical physics. *Rev. Mod. Phys.* **85**, 1115–1141 (2013).
9. Jenkinson, G., Abante, J., Feinberg, A. P. & Goutsias, J. An information-theoretic approach to the modeling and analysis of whole-genome bisulfite sequencing data. *BMC Bioinformatics* **19**, 87 (2018).
10. Baxter, R. J. Exactly Solved Models in Statistical Mechanics (Academic Press, San Diego, 1982).
11. Zhan, Z.-H., Zhang, J., Li, Y. & Chung, H. S.-H. Adaptive particle swarm optimization. *IEEE T. Syst. Man Cy. B* **39**, 1362–1381 (2009).
12. Jones, D. R. Direct global optimization algorithm. In Encyclopedia of Optimization, C. A. Floudas and P. M. Pardalos, eds (Springer, Boston, 2001).
13. Huyer, W. & Neumaier, A. Global optimization by multilevel coordinate search. *J. Global Optim.* **14**, 331–355 (1999).

14. Goffe, W. L., Ferrier, G. D. & Rogers, J. Global optimization of statistical functions with simulated annealing. *J. Econom.* **60**, 65–99 (1994).

15. Spall, J. C. Introduction to Stochastic Search and Optimization: Estimation, Simulation, and Control (Wiley-Interscience, Hoboken, 2003).

16. Glasmachers, T., Schaul, T., Yi, S., Wierstra, D. & Schmidhuber, J. Exponential natural evolution strategies. Proceedings of the 12th Annual Conference on Genetic and Evolutionary Computation, Genetic and Evolutionary Computation Conference, Portland, Oregon, USA, July 7-11, 393–400 (2010).

17. Kolda, T. G., Lewis, R. M. & Torczon, V. Optimization by direct search: New perspectives on some classical and modern methods. *SIAM Rev.* **45**, 385–482 (2003).

18. Mogensen, P. K. & Riseth, A. N. Optim: A mathematical optimization package for Julia. *J. Open Source Softw.* **3**, 615 (2018).

19. Bezanson, J., Edelman, A., Karpinski, S. & Shah, V. B. Julia: A fresh approach to numerical computing. *SIAM Rev.* **59**, 65–98 (2017).

20. Bickel, P. J. & Doksum, K. A. Mathematical Statistics: Basic Ideas and Selected Topics, vol. I (CRC Press, Boca Raton, 2015).

21. Benjamini, Y. & Hochberg, Y. Controlling the false discovery rate: a practical and powerful approach to multiple testing. *J. R. Stat. Soc. B* **57**, 289–300 (1995).

22. Marozzi, M. Some remarks about the number of permutations one should consider to perform a permutation test. *Statistica* **64**, 193–201 (2004).

23. Gu, H. et al. Preparation of reduced representation bisulfite sequencing libraries for genome-scale DNA methylation profiling. *Nat. Protocols* **6**, 468–481 (2011).

24. Langmead, B. Aligning short sequencing reads with Bowtie. *Curr. Protoc. Bioinformatics* Chapter 11, Unit–11.7 (2010).

25. Krueger, F. & Andrews, S. R. Bismark: a flexible aligner and methylation caller for bisulfite-seq applications. *Bioinformatics* **27**, 1571–1572 (2011).
