# Supplementary material for: Detection of haplotype-dependent allele-specific DNA methylation in WGBS data
Source: Nat Commun. 2020 Oct 16;11:5238. doi: 10.1038/s41467-020-19077-1 (PMC7567826; doi:10.1038/s41467-020-19077-1)
Supplement: Supplementary file 3 — Reporting Summary [file 41467_2020_19077_MOESM3_ESM.pdf]

nature research

Corresponding author(s): John Goutsias

Last updated by author(s): August 26, 2020

Reporting Summary

Nature Research wishes to improve the reproducibility of the work that we publish. This form provides structure for consistency and transparency in reporting. For further information on Nature Research policies, see our Editorial Policies and the Editorial Policy Checklist.

Statistics

For all statistical analyses, confirm that the following items are present in the figure legend, table legend, main text, or Methods section.

n/a	Confirmed	
☐	☒	The exact sample size (n) for each experimental group/condition, given as a discrete number and unit of measurement
☐	☒	A statement on whether measurements were taken from distinct samples or whether the same sample was measured repeatedly
☐	☒	The statistical test(s) used AND whether they are one- or two-sided<br>*Only common tests should be described solely by name; describe more complex techniques in the Methods section.*
☒	☐	A description of all covariates tested
☐	☒	A description of any assumptions or corrections, such as tests of normality and adjustment for multiple comparisons
☐	☒	A full description of the statistical parameters including central tendency (e.g. means) or other basic estimates (e.g. regression coefficient) AND variation (e.g. standard deviation) or associated estimates of uncertainty (e.g. confidence intervals)
☐	☒	For null hypothesis testing, the test statistic (e.g. F, t, r) with confidence intervals, effect sizes, degrees of freedom and P value noted<br>*Give P values as exact values whenever suitable.*
☒	☐	For Bayesian analysis, information on the choice of priors and Markov chain Monte Carlo settings
☒	☐	For hierarchical and complex designs, identification of the appropriate level for tests and full reporting of outcomes
☒	☐	Estimates of effect sizes (e.g. Cohen's d, Pearson's r), indicating how they were calculated

Our web collection on statistics for biologists contains articles on many of the points above.

Software and code

Policy information about availability of computer code

Data collection	Trim Galore (v0.5.0), Arioc (v1.3.0), Picard (v2.18), Bismark (v0.20.0), SNPsplit (v0.3.2), WhatsHap (v0.17)
Data analysis	CpelAsm accessible at https://github.com/jordiabante/CpelAsm.jl.

For manuscripts utilizing custom algorithms or software that are central to the research but not yet described in published literature, software must be made available to editors and reviewers. We strongly encourage code deposition in a community repository (e.g. GitHub). See the Nature Research guidelines for submitting code & software for further information.

Data

Policy information about availability of data

All manuscripts must include a data availability statement. This statement should provide the following information, where applicable:

- Accession codes, unique identifiers, or web links for publicly available datasets
- A list of figures that have associated raw data
- A description of any restrictions on data availability

The raw WGS and WGBS data are available from NIH's Epigenomics Roadmap Initiative with accession number PRJNA34535 for patient ID STL003 [https://www.ncbi.nlm.nih.gov/bioproject/?term=PRJNA34535]. The raw SNP data can be downloaded from Genboree with patient ID STL003 [ftp://ftp.genboree.org/allelic-epigenome/json]. The homo sapiens (human) genome assembly GRCh37 (hg19) used as a reference genome can be downloaded from [https://hgdownload.soe.ucsc.edu/goldenPath/hg19/bigZips/]. The gene imprinting data can be downloaded from [https://www.geneimprint.com/site/genes-by-species.Homo+sapiens.imprinted-All]. The enhancer data can be downloaded from [https://personal.broadinstitute.org/meuleman/reg2map/HoneyBadger2-intersect_release/DNase/p10/enh/BED_files_per_cluster].

Field-specific reporting

Please select the one below that is the best fit for your research. If you are not sure, read the appropriate sections before making your selection.

☒ Life sciences ☐ Behavioural & social sciences ☐ Ecological, evolutionary & environmental sciences

For a reference copy of the document with all sections, see nature.com/documents/nr-reporting-summary-flat.pdf

Life sciences study design

All studies must disclose on these points even when the disclosure is negative.

Sample size	The sample size varied in each sample for each haplotype allele analyzed depending on the number of available sequencing reads. The minimum sample size used in this study was five observations per haplotype allele, which ensures that the parameter estimation is reliable and ensures control of the type I error.
Data exclusions	Haplotypes that did not meet the minimum coverage requirements were excluded from the analysis due to insufficient data for model estimation (see Supplementary Methods). This was a pre-established criteria based on simulation results. Within each sample processed, different numbers of haplotypes were processed due to different coverages, ranging from 45,162 to 210,584 out of a total number of 715,155 haplotypes defined in each sample.
Replication	Reproducibility of experimental findings is not an issue here, since this is not an experimental paper. However, a minimum of 1,000 null statistics were used to construct a null distribution via bootstrapping and use it to perform hypothesis testing on each haplotype analyzed, which ensures reproducibility of the allele-specific methylation analysis performed (see Supplementary Information).
Randomization	Not relevant since the study is not experimental and only uses data from a single subject.
Blinding	Not relevant since the study is not experimental and only uses data from a single subject who was not exposed to any intervention.

Reporting for specific materials, systems and methods

We require information from authors about some types of materials, experimental systems and methods used in many studies. Here, indicate whether each material, system or method listed is relevant to your study. If you are not sure if a list item applies to your research, read the appropriate section before selecting a response.

Materials & experimental systems

n/a	Involved in the study	
☒	☐	Antibodies
☒	☐	Eukaryotic cell lines
☒	☐	Palaeontology and archaeology
☒	☐	Animals and other organisms
☒	☐	Human research participants
☒	☐	Clinical data
☒	☐	Dual use research of concern

Methods

n/a	Involved in the study	
☒	☐	ChIP-seq
☒	☐	Flow cytometry
☒	☐	MRI-based neuroimaging
